# Neighborhood Socioeconomic Disadvantage and White Matter Microstructure of the Arcuate Fasciculus and Uncinate Fasciculus in Adolescents

**DOI:** 10.1016/j.bpsgos.2023.10.002

**Published:** 2023-10-14

**Authors:** Artenisa Kulla, Saché Coury, Jordan M. Garcia, Giana I. Teresi, Lucinda M. Sisk, Melissa Hansen, Jonas G. Miller, Ian H. Gotlib, Tiffany C. Ho

**Affiliations:** a College of Medicine, University of Florida, Gainesville, Florida; b Department of Psychology, Stanford University, Stanford, California; c Department of Psychology, University of Pittsburgh, Pittsburgh, Pennsylvania; d Department of Psychology, Yale University, New Haven, Connecticut; e Department of Psychology, Colorado State University, Fort Collins, Colorado; f Department of Psychological Sciences, University of Connecticut, Storrs, Connecticut; g Department of Psychology, University of California, Los Angeles, Los Angeles, California

**Keywords:** Adolescence, Arcuate fasciculus, Poverty, Socioeconomic status, Uncinate fasciculus

## Abstract

**Background:**

Neighborhood- or area-level socioeconomic disadvantage is associated with neural alterations across the life span. However, few studies have examined the effects of neighborhood disadvantage on white matter microstructure during adolescence, an important period of development that coincides with increased risk for psychopathology.

**Methods:**

In 200 adolescents (ages 13–20 years; 54.5% female, 4% nonbinary) recruited from 2 studies enriched for early adversity and depression, we examined whether neighborhood socioeconomic disadvantage derived from census tract data was related to white matter microstructure in several major white matter tracts. We also examined whether depressive symptoms and sex moderated these associations.

**Results:**

Greater neighborhood socioeconomic disadvantage was associated with lower fractional anisotropy (FA) in the left arcuate fasciculus (β = −0.24, false discovery rate [FDR]–corrected *p* = .035) and right uncinate fasciculus (β = −0.32, FDR-corrected *p* = .002) above and beyond the effects of family-level socioeconomic status. Depressive symptoms significantly moderated the association between left arcuate fasciculus FA and both neighborhood (β = 0.17, FDR-corrected *p* = .026) and unemployment (β = 0.22, FDR-corrected *p* = .004) disadvantage such that these associations were only significant in adolescents who reported less severe depression. Sex did not moderate the association between socioeconomic disadvantage and FA in these tracts.

**Conclusions:**

Greater neighborhood socioeconomic disadvantage, particularly poverty and educational attainment levels, was associated with lower FA in the arcuate fasciculus and uncinate fasciculus above and beyond the effects of family-level measures of socioeconomic status. These patterns were only observed in adolescents with low levels of depression, suggesting that we must be cautious about generalizing these findings to youths who struggle with mental health difficulties.

Socioeconomic disadvantage is one of the strongest predictors of difficulties in physical and mental well-being, with growing evidence that youths are vulnerable to the enduring consequences of low socioeconomic status (SES), broadly defined (1). In the context of mental health, socioeconomically disadvantaged children and adolescents have been found to be twice as likely as their advantaged peers to develop mental health disorders, especially if the disadvantage is chronic (2). Socioeconomic disadvantage is a complex and multifaceted construct that can be examined at multiple levels. Research that has examined factors related to SES and the developing brain has generally focused on family-level indices of advantage (3,4). However, measuring disadvantage at the neighborhood or area level is critical to capture the larger social context that children are exposed to (e.g., structural inequities, community resources, pollutants) that are not insufficiently captured by household-level measures of SES [see (5)]. In these studies, family-level SES—which has typically been operationalized as the highest level of parental education and/or household income achieved—has been found to be positively associated with cortical surface area in a variety of brain regions (3,6,7). Furthermore, despite being more distal to an individual, neighborhood- or community-level contexts—including neighborhood violence, poverty rates, and unemployment rate, among others—confer additional risk beyond family-level factors because they can increase exposure to other adverse experiences and limit access to material or social support, particularly among adolescents who are gaining independence in their development and thus are not limited to the exposures found in their immediate home (8, 9, 10).

Recent work has shown that neighborhood socioeconomic disadvantage, derived from census tract data, affects brain morphometry in adolescence. Specifically, youths living in less advantaged communities exhibited thinner global and regional cortices in the left hemisphere; in contrast, family-level SES factors were not related to global patterns of cortical thickness (11). Other studies have implicated neighborhood socioeconomic disadvantage in how the brain integrates information, highlighting faster functional brain development in advantaged than in disadvantaged adolescents (12). Therefore, socioeconomic disadvantage has been broadly associated with gray matter morphometry; relatively fewer studies have examined associations with white matter development (4).

Given that experience-dependent myelination is the primary process that drives neuroplasticity during childhood and adolescence (13), it is critical to elucidate how socioeconomic disadvantage affects white matter during this sensitive developmental period. Disparities in family-level SES have been implicated in white matter organization in children, especially in tracts that support executive functioning, cognitive control, and language processing, such as the cingulum cingulate (CC), inferior longitudinal fasciculus, and corticospinal tract (CST) (7). In these studies, lower family-level SES have consistently been associated with smaller values of fractional anisotropy (FA) that are indicative of aberrant white matter microstructure. However, it is not clear whether this pattern holds for neighborhood-level socioeconomic disadvantage and which specific SES factors (e.g., poverty, housing burden) have the strongest neural consequences. Bell *et al.* (14) recently examined the impact of neighborhood disadvantage—operationalized as a composite of neighborhood poverty, education, unemployment, race, income, and home ownership—on white matter microstructure implicated in emotional functioning in 303 young adults (mean age = 20 years). Bell *et al.* (14) reported that lower white matter microstructure, as indexed by quantitative anisotropy, in fronto-cingulate-limbic tracts (including the uncinate fasciculus [UF] and cingulum bundles) was associated with greater neighborhood disadvantage. Thus, white matter pathways that support emotional functioning appear to be adversely affected by the level of resources in the environment accessible at the neighborhood level, that is, beyond the participant’s immediate home environment. Because adolescence is widely considered to be a sensitive period of neurodevelopment in that environmental input experienced during this period may exert a greater influence on subsequent outcomes (13,15), it is critical to examine whether these patterns of white matter microstructure are also present in adolescents specifically, whether there are effects outside of the limited tracts examined in the investigation by Bell *et al.* (14), and whether specific indicators that constitute neighborhood-level disadvantage have distinct effects on various tracts given their differences in developmental trajectories (16).

Moreover, adolescents who experience socioeconomic disadvantage also experience more mental health difficulties (2,17). However, how mental health problems may moderate associations between disadvantage and white matter tract integrity is less understood. Previous studies have independently identified neural changes associated with adversity (4,6) and depression (18, 19, 20), suggesting that youths who both have depression and experience disadvantage may demonstrate differential neural characteristics. From a cumulative risk perspective, we would hypothesize that adolescents with mental health difficulties will show a stronger effect of neighborhood disadvantage on brain phenotypes such as myelination through stress processes (e.g., inflammation, cortisol) (13). Alternatively, changes in the brain that arise from mental health difficulties could alter mechanisms of plasticity that limit the extent to which broader environmental influences—for better or worse—influence subsequent brain development (21). Therefore, testing the role of mental health symptoms as a potential moderator of the association between neighborhood disadvantage and adolescent brain maturation is needed to explore this possibility.

It is also important to consider potential sex differences in the associations among neighborhood socioeconomic disadvantage, brain development, and mental health. For example, Leventhal and Brooks-Gunn (22) found that as neighborhood conditions improved (e.g., private housing, lower poverty levels), young boys, but not young girls, had significantly lower levels of depression and anxiety. More recently, King *et al.* (23) found that adolescents who lived in disadvantaged neighborhoods, measured by neighborhood poverty levels, had higher levels of depression and anxiety than their advantaged peers and, furthermore, that this effect was specific to girls. Considering recent evidence that there are sex-specific effects of depression on myelin content in adolescents (24), it is important that we investigate the specific interactions of neighborhood disadvantage, sex, and the developing brain in the context of mental health. It is also important to examine whether those who are experiencing mental health difficulties, particularly young adolescent girls who are at greater risk than their male peers, are characterized by stronger associations between neighborhood disadvantage and brain development; doing so will inform screening and intervention in youths.

To address these questions, we examined the effects of neighborhood-level socioeconomic disadvantage on FA across 2 independent cohorts of adolescents who were comprehensively characterized with respect to their exposure to early adversity (a potent risk factor for depression) or on severity of depression. Specifically, we examined relationships between census tract data indexing socioeconomic disadvantage and individuals’ white matter tract integrity to test whether neighborhood-level disadvantage is related to FA in white matter tracts that support executive functioning, cognitive control, emotion processing, and language development (7,14,25,26) and that have also been implicated in adolescent depression (19,24): the arcuate fasciculus (AF), CC, CST, inferior longitudinal fasciculus, and UF. The decision to investigate these tracts was informed in part by Bell *et al.*’s study (14). In post hoc analyses, we examined which individual indicators (educational attainment, poverty, unemployment, housing burden, and linguistic isolation) explained our findings. Then, we tested whether severity of depression moderated these effects. Based on previous literature, we hypothesized that greater neighborhood disadvantage would be associated with lower FA in all white matter tracts of interest and that severity of depression would amplify these effects such that greater neighborhood disadvantage would be associated with lower FA in these tracts in adolescents with more severe depression. Finally, in exploratory analyses, we examined whether there were sex differences in any of our statistically significant models.

## Methods and Materials

### Participants

Data from the current study were collected through 2 ongoing longitudinal neuroimaging studies at Stanford University: the TIGER (Teen Inflammation Glutamate Emotion Research) study (27) (National Institutes of Health Grant No. K01MH117442) and the ELS (Early Life Stress) study (National Institutes of Health Grant No. R37MH101495). Data from both cohorts were collected between 2017 and 2021. Because the primary goal of the TIGER study was to compare adolescents with depression and healthy control adolescents, inclusion/exclusion criteria differed for these groups. Participants for the ELS study were recruited as part of a 4-wave longitudinal study characterizing the effects of early-life stress on brain development across the pubertal transition (28,29). In the current investigation, we included data from the third wave of the ELS study, when participants were ages 14 to 17 years, because the ages and pubertal stages of the ELS participants during this period of time were comparable to those of the adolescents participating in the TIGER study. See the Supplement for more details on inclusion/exclusion criteria. In accordance with the Declaration of Helsinki, all participants provided informed assent, and their parent(s)/legal guardian(s) provided informed consent. All participants were compensated for study participation with gift cards. TIGER was approved by the Institutional Review Boards at the University of California, San Francisco and Stanford University, and ELS was approved by the Institutional Review Board at Stanford University.

Of the 262 participants (93 TIGER, 169 ELS) who met eligibility criteria and underwent magnetic resonance imaging (MRI) scanning, 58 were excluded due to excessive motion during the diffusion MRI scan, 1 was excluded due to a coverage error during acquisition, and 1 was excluded due to a brain anomaly observed in their anatomical scan. Of the remaining 202 participants who provided an address for us to obtain census tract data, 2 lived outside the state of California and thus were excluded from analysis. One participant resided in 2 California ZIP codes during study participation, so we used the ZIP code with the longest residence history. The excluded participants did not differ significantly from those who were included on any demographic variable (all *p*s > .071). In total, we included data from 200 participants in the present analysis (78 TIGER, 122 ELS).

### Neuroimaging Acquisition

All but 47 participants (9 TIGER, 38 ELS) were scanned on a 3T Discovery MR750 (GE Medical Systems) with a 32-channel head coil (Nova Medical) at the Stanford Center for Cognitive Neuroscience and Neurobiological Imaging located in the Department of Psychology. The remaining 47 participants were assessed after a scanner hardware upgrade to SIGNA Ultra High Performance that coincided with the period of time when COVID-19 mitigation procedures were put in place; thus, in all statistical analyses, scan time point (pre-COVID/scanner upgrade vs. post-COVID/scanner upgrade) was included as a binary covariate. Participant height and weight were measured at the conclusion of the scan to calculate body mass index. See the Supplement for more details on the acquisition parameters for each scan.

### Deterministic Tractography Using Automated Fiber Quantification

Diffusion MRI data were processed using the open-source mrVista software distribution developed by the VISTA lab (https://vistalab.stanford.edu/). Streamlines in each of the tracts of interest—the bilateral AF, CC, CST, inferior fronto-occipital fasciculus (IFOF), and UF—were automatically generated using a 2-planar waypoint region of interest approach (30). All tracts were visually inspected by the first and senior authors for consistency. Because automated fiber quantification computes diffusivity metrics for 100 evenly spaced nodes along the tract, we averaged FA along the entire tract for a more reliable estimate, as in our previous work (24,31).

### Neighborhood Disadvantage Data

Neighborhood disadvantage percentile scores were extracted based on census tract data from the California Communities Environmental Screening tool (CalEnviroScreen 3.0) released by the California Environmental Protection Agency (https://oehha.ca.gov/calenviroscreen/report/calenviroscreen-30) according to participant’s address and ZIP code at the time of the neuroimaging scan. The CalEnviroScreen 3.0 provides a composite index of neighborhood disadvantage. Specifically, the composite index score, called population characteristics, was derived from average percentiles of public health indicators and socioeconomic indicators. The socioeconomic indicators, which were of key interest, included the following: educational attainment, poverty, housing burden, linguistic isolation, and unemployment. See the Supplement for more details on how the percentiles for each indicator were calculated. In our sample (*N* = 200), a total of 203 census tracts and 112 ZIP codes were represented. A maximum of 5 participants were living in 1 census tract and a maximum of 10 were living in 1 ZIP code. In supplemental analyses, we also reran all significant models with data from the CalEnviroScreen4.0 data, which was released in 2021 and covers the time periods 3 years after the 3.0 release (and, for many of the participants in our study, years after data collection).

### Depression Severity

Adolescents completed the Reynolds Adolescent Depression Scale (RADS-2), a 30-item scale validated in youths ages 11 to 20 years (32). A RADS-2 score of 75 is considered the clinical cutoff for depression (with a score of 76–81 indicating levels of mild depression). In both studies, the RADS-2 was administered approximately 2 weeks before the neuroimaging scan (mean: 15.4 days).

### Statistical Analyses

All statistical analyses were conducted using R version 4.2.3 for MacOS Monterey (see Key Resources Table). Study groups were compared on demographic metrics using Student’s *t* tests and χ^2^ tests, where appropriate. We used linear regression models to examine the following specific hypotheses: 1) there would be associations between a composite score of neighborhood disadvantage and FA in the tracts of interest across the entire sample; 2) there would be distinct associations of each of the 5 socioeconomic disadvantage indicators that comprise the composite neighborhood disadvantage score with FA in the tracts of interest across the entire sample; 3) there would be a moderating effect of depression severity (RADS-2) on associations between each of the 5 socioeconomic disadvantage indicators and the composite score and the tracts of interest; and 4) there would be a moderating effect of sex on associations between neighborhood disadvantage and subsequent indicators and the tracts of interest. For models that yielded a statistically significant effect of moderation by sex, we also conducted our analyses stratified by sex (i.e., within boys and girls separately). Statistical assumptions of the linear regression models (positive predictor check, linearity and collinearity, normality of residuals, homogeneity of variance, and the presence of potentially influential observations) were checked via diagnostic plots and tables using the check_model function in the package *performance* and nice_assumptions function in the package *rempsyc*.

In all primary statistical analyses, we included age, sex, body mass index, tract length, family-level SES (highest level of parental educational attainment), study group (TIGER/ELS), race (American Indian or Alaska Native, Asian, Black or African American, Native Hawaiian or other Pacific Islander, White, multiracial, or other), psychiatric medication use (yes/no), RADS-2 total score (where appropriate), scan time point (pre- vs. post-COVID), and motion (a single value averaged across all 6 axes) during the diffusion-weighted MRI scan as covariates. We also include all models run without covariates in the Supplement. False discovery rate (FDR) correction was applied for given tracts of interest per hemisphere (i.e., left- and right-lateralized tracts were corrected for separately). Finally, more advanced pubertal staging was positively associated with depressive symptoms (*r* = 0.17, *p* = .022); therefore, in models in which depression severity was tested as a moderator, we included Tanner score as a covariate. See Figure S1 for the distribution of Tanner scores in our sample.

## Results

### Participant Characteristics

Demographic and clinical characteristics of the participants are presented in Table 1. As expected, the participants from the TIGER study reported a significantly higher severity of depression, measured by RADS-2 scores, and greater use of psychiatric medications (all *p*s < .001). In addition, a higher percentage of participants in the ELS than in the TIGER study were scanned after the COVID scanner upgrade. ELS participants also experienced greater neighborhood disadvantage overall and with respect to education, poverty, unemployment, and housing burden (*p*s < .023). Poverty and educational attainment levels were also correlated with one another (*r* = 0.81, *p* < .001) and highly collinear in our models (variance inflation factors > 3), and therefore it was necessary to parse the independent contributions of these components. Importantly, however, the 2 study groups did not differ on any potentially confounding demographic variables, use of nonpsychiatric medications, length of any tract of interest, or motion during the scan (all *p*s > .066). See Table S1 for more details.

**Table 1:** Descriptive Statistics for Demographic and Primary Variables of Interest in the Final Analytic Sample (*N* = 200)

Variables of Interest	Total, *N* = 200
Age, Years	
Mean (SD)	15.927 (1.290)
Range	13.065–20.080
Sex	
Female	115 (57.5%)
Male	85 (42.5%)
Gender	
Female	109 (54.5%)
Male	83 (41.5%)
Nonbinary	8 (4.0%)
Ethnicity	
Hispanic or Latino	28 (14.0%)
Non-Hispanic or Latino	172 (86.0%)
Race	
American Indian or Alaska Native	5 (2.5%)
Asian	36 (18.0%)
Black or African American	12 (6.0%)
Multiracial	32 (16.0%)
Native Hawaiian or Other Pacific Islander	0 (0.0%)
Other	20 (10.0%)
White	95 (47.5%)
Scanned During COVID-19	
No	153 (76.5%)
Yes	47 (23.5%)
Tanner Score	
Missing	7
Mean (SD)	4.415 (0.581)
Range	2.000–5.000
Parental Level of Education	
Missing	6
Less than a high school diploma	0 (0%)
High school graduate or equivalent (GED)	3 (1.5%)
Some college, no degree	21 (10.8%)
Associate’s degree (e.g., A.A., A.S.)	10 (5.2%)
Bachelor’s degree (e.g., B.A., B.S.)	52 (26.8%)
Master’s degree (e.g., M.A., M.S., M.Ed.)	79 (40.7%)
Doctoral or professional degree (M.D., D.D.S., D.V.M., Ph.D., Ed.D.)	29 (14.9%)
Psychiatric Medication Status	
No medication use	162 (81.0%)
Medication use	38 (19.0%)
Corticosteroid Use	
Missing	8
No corticosteroid use	178 (92.7%)
Corticosteroid use	14 (7.3%)
Body Mass Index	
Missing	1
Mean (SD)	22.109 (4.665)
Range	14.747–39.247
Diagnostic History of Major Depressive Disorder	
No	111 (55.5%)
Yes	89 (44.5%)
RADS-2 Total Score	
Missing	7
Mean (SD)	64.135 (17.767)
Range	30.000–112.000
Education Percentile Score	
Missing	3
Mean (SD)	27.005 (21.666)
Range	0.040–86.270
Poverty Percentile Score	
Mean (SD)	21.499 (21.785)
Range	0.030–84.500
Unemployment Percentile Score	
Mean (SD)	26.226 (20.837)
Range	0.360–89.910
Housing Burden Percentile Score	
Missing	1
Mean (SD)	30.179 (23.190)
Range	0.130–91.700
Linguistic Isolation Percentile Score	
Missing	2
Mean (SD)	44.116 (23.868)
Range	0.000–94.410
Population Characteristics Percentile Score	
Missing	1
Mean (SD)	24.858 (22.525)
Range	0.030–93.870
Left AF FA Mean	
Missing	2
Mean (SD)	0.493 (0.032)
Range	0.384–0.584
Left AF Tract Length, mm	
Mean (SD)	13,187.357 (48,835.153)
Range	866.110–330,000.000
Right AF FA Mean	
Missing	24
Mean (SD)	0.468 (0.034)
Range	0.363–0.554
Right AF Tract Length, mm	
Missing	2
Mean (SD)	3685.867 (9151.926)
Range	631.290–94,949.000
Left UF FA Mean	
Missing	3
Mean (SD)	0.437 (0.032)
Range	0.348–0.538
Left UF Tract Length, mm	
Missing	1
Mean (SD)	3864.351 (2312.650)
Range	1167.700–16,480.000
Right UF FA Mean	
Mean (SD)	0.433 (0.029)
Range	0.340–0.502
Right UF Tract Length, mm	
Mean (SD)	2341.941 (2012.800)
Range	584.730–19,304.000
Left Corticospinal FA Mean	
Missing	1
Mean (SD)	0.638 (0.024)
Range	0.580–0.712
Left Corticospinal Tract Length, mm	
Missing	1
Mean (SD)	2641.001 (1469.837)
Range	1145.000–10,183.000
Right Corticospinal FA Mean	
Missing	1
Mean (SD)	0.621 (0.026)
Range	0.547–0.691
Right Corticospinal Tract Length, mm	
Missing	1
Mean (SD)	1693.488 (978.769)
Range	421.060–8001.200
Left Cingulum Cingulate FA Mean	
Missing	4
Mean (SD)	0.507 (0.044)
Range	0.352–0.609
Left Cingulum Cingulate Tract Length, mm	
Missing	2
Mean (SD)	3786.420 (2484.077)
Range	938.920–18,612.000
Right Cingulum Cingulate FA Mean	
Missing	2
Mean (SD)	0.469 (0.045)
Range	0.336–0.613
Right Cingulum Cingulate Tract Length, mm	
Mean (SD)	3497.078 (2759.722)
Range	554.820–29,447.000
Left IFOF FA Mean	
Mean (SD)	0.490 (0.029)
Range	0.419–0.567
Left IFOF Tract Length, mm	
Mean (SD)	6451.111 (3734.863)
Range	1851.200–22,823.000
Right IFOF FA Mean	
Mean (SD)	0.492 (0.027)
Range	0.415–0.557
Right IFOF Tract Length, mm	
Mean (SD)	3871.771 (2509.980)
Range	1599.600–18,954.000
Motion During Scan	
Mean (SD)	−0.052 (0.059)
Range	−0.217 to 0.126

Values are presented as *n* or *n* (%) unless indicated otherwise. Motion refers to the average amount of movement across the 6 axes during the diffusion magnetic resonance imaging scan, where negative values refer to displacement in the leftward direction for x, the posterior direction for y, the inferior direction for z, leftward tilt for pitch, counterclockwise rotation for roll, and downward tilt for yaw.

AF, arcuate fasciculus; FA, fractional anisotropy; GED, General Educational Development; IFOF, inferior fronto-occipital fasciculus; RADS-2, Reynolds Adolescent Depression Scale; UF, uncinate fasciculus.

### Higher Percentiles of Neighborhood Disadvantage Are Associated With Lower FA in the AF and UF

**Figure 1:**
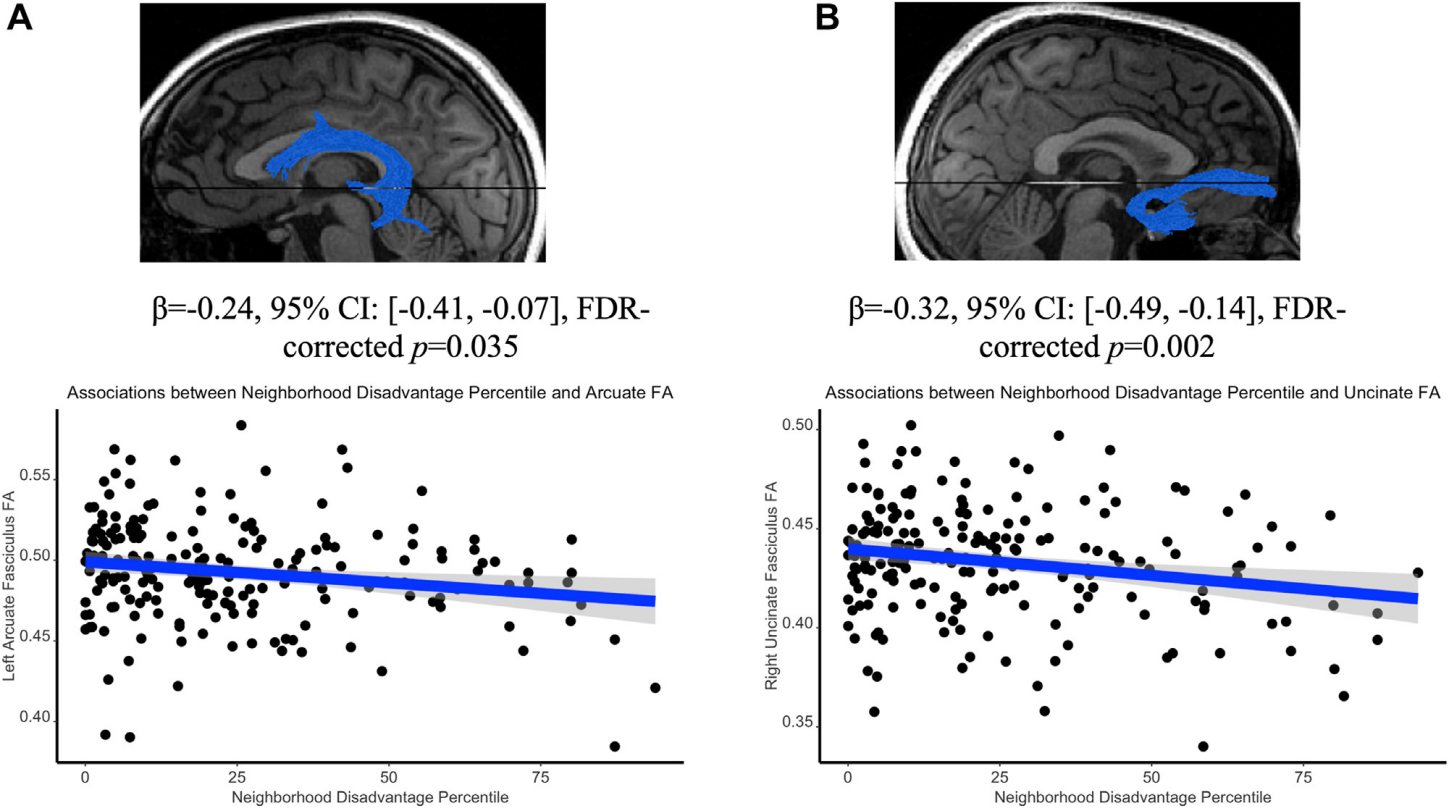
Significant linear associations between neighborhood disadvantage percentile and fractional anisotropy (FA) of the arcuate fasciculus **(A)** and uncinate fasciculus **(B)**. All data are displayed without adjustment for covariates for visualization purposes only. See Table 2 for more details. FDR, false discovery rate.

We tested whether neighborhood disadvantage percentiles were associated with FA in the tracts of interest across the entire sample. When accounting for covariates, we found that higher percentiles of neighborhood disadvantage, measured by the population characteristics composite score, were significantly associated with lower FA in the bilateral AF and right UF. After applying FDR correction based on the number of tracts in each hemisphere, the strongest effects that survived were found in the left AF (β = −0.24, 95% CI, −0.41 to −0.07, FDR-corrected *p* = .035) and right UF (β = −0.32, 95% CI, −0.49 to −0.14, FDR-corrected *p* = .002). See Figure 1 and Table 2. FA in all other tracts (CST, IFOF, and CC) was not significantly associated with neighborhood disadvantage percentiles (all FDR-corrected *p*s > .108). See Table 2. These results did not change even without covariate adjustment (Tables S2A, B).

**Table 2:** Summary of Estimated Linear Associations Between Neighborhood Disadvantage Percentile and Tract FA in Left and Right Hemispheres

Tract	Beta Coefficient	SE	95% CI	*t* Value	*p* Value	FDR-Corrected *p* Value	*R*^2^	Δ*R*^2^
Left Hemisphere								
Left AF FA	−0.24	0.09	−0.41 to −0.07	−2.74	.007^a^	.035	0.164	0.04
Left CC FA	−0.14	0.08	−0.31 to 0.03	−1.67	.096	.160	0.213	0.02
Left CST FA	−0.08	0.08	−0.24 to 0.09	−0.89	.377	.377	0.160	0.01
Left IFOF FA	−0.13	0.09	−0.30 to 0.04	−1.51	.134	.168	0.141	0.01
Left UF FA	−0.16	0.09	−0.34 to 0.01	−1.85	.066	.160	0.147	0.02
Right Hemisphere								
Right AF FA	−0.2	0.10	−0.39 to −0.01	−2.04	.043^b^	.108	0.102	0.03
Right CC FA	−0.02	0.09	−0.19 to 0.16	−0.19	.846	.846	0.133	0.0002
Right CST FA	−0.13	0.08	−0.29 to 0.03	−1.59	.114	.19	0.255	0.02
Right IFOF FA	−0.05	0.09	−0.22 to 0.11	−0.63	.528	.66	0.196	0.002
Right UF FA	−0.32	0.09	−0.49 to −0.14	−3.60	<.001^a^	.002	0.142	0.073

In all linear models, age, sex, body mass index, depression severity, psychiatric medication use, study group, race, scan time point, tract length, motion during the scan, and parental education level were included as covariates. All reported beta coefficients are standardized.

AF, arcuate fasciculus; CC, cingulum cingulate; CST, corticospinal tract; FA, fractional anisotropy; FDR, false discovery rate; IFOF, inferior fronto-occipital fasciculus; UF, uncinate fasciculus.

a *p* < .01.

b *p* < .05.

Meanwhile, when examining the association between parental education level and FA in the tracts of interest, we found that lower parental education level was significantly associated with lower FA in the left UF. However, this association did not survive FDR correction (β = −1.29, 95% CI, −2.52 to −0.05, FDR-corrected *p* = .205). Parental education was not associated with FA in any other tracts (all FDR-corrected *p*s > .205). These results remained nonsignificant without covariate adjustment (Tables S3A–D).

### Post Hoc Analyses: Educational Attainment and Poverty Are Associated With FA of the AF and UF

In post hoc analyses, we tested which of the 5 socioeconomic factor indicators (educational attainment, poverty, unemployment, housing burden, and linguistic isolation) that comprised the neighborhood disadvantage score were driving the association with FA in the AF and UF tracts across the entire sample. In a covariate-adjusted model, higher education disadvantage percentiles were significantly associated with lower FA in the bilateral AF, left CC, and left IFOF; the strongest effects that survived FDR correction were found in the left AF (β = −0.22, 95% CI, −0.38 to −0.06, FDR-corrected *p* = .028), left CC (β = −0.21, 95% CI, −0.37 to −0.05, FDR-corrected *p* = .028), and left IFOF (β = −0.19, 95% CI, −0.36 to −0.03, FDR-corrected *p* = .04). Higher poverty percentiles were significantly associated with lower FA in the left AF (β = −0.21, 95% CI, −0.37 to −0.06, FDR-corrected *p* = .02) and bilateral UF (left UF: β = −0.24, 95% CI, −0.39 to −0.08, FDR-corrected *p* = .015; right UF: β = −0.26, 95% CI, −0.42 to −0.10, FDR-corrected *p* = .01). None of the other socioeconomic factor indicators, including housing burden and linguistic isolation, were associated with FA in any of the tracts (FDR-corrected *p*s > .05). See Tables S4–S8 and Figure S2. We also report these models without covariate adjustment in Tables S4B, E, S5B, E, S6B, D, S7B, D, and S8B, D. These results largely did not change with CalEnviroScreen4.0 data (Tables S4–S8).

### Post Hoc Analysis: Depression Severity Moderates the Association Between Neighborhood Disadvantage and FA of the Left AF

**Figure 2:**
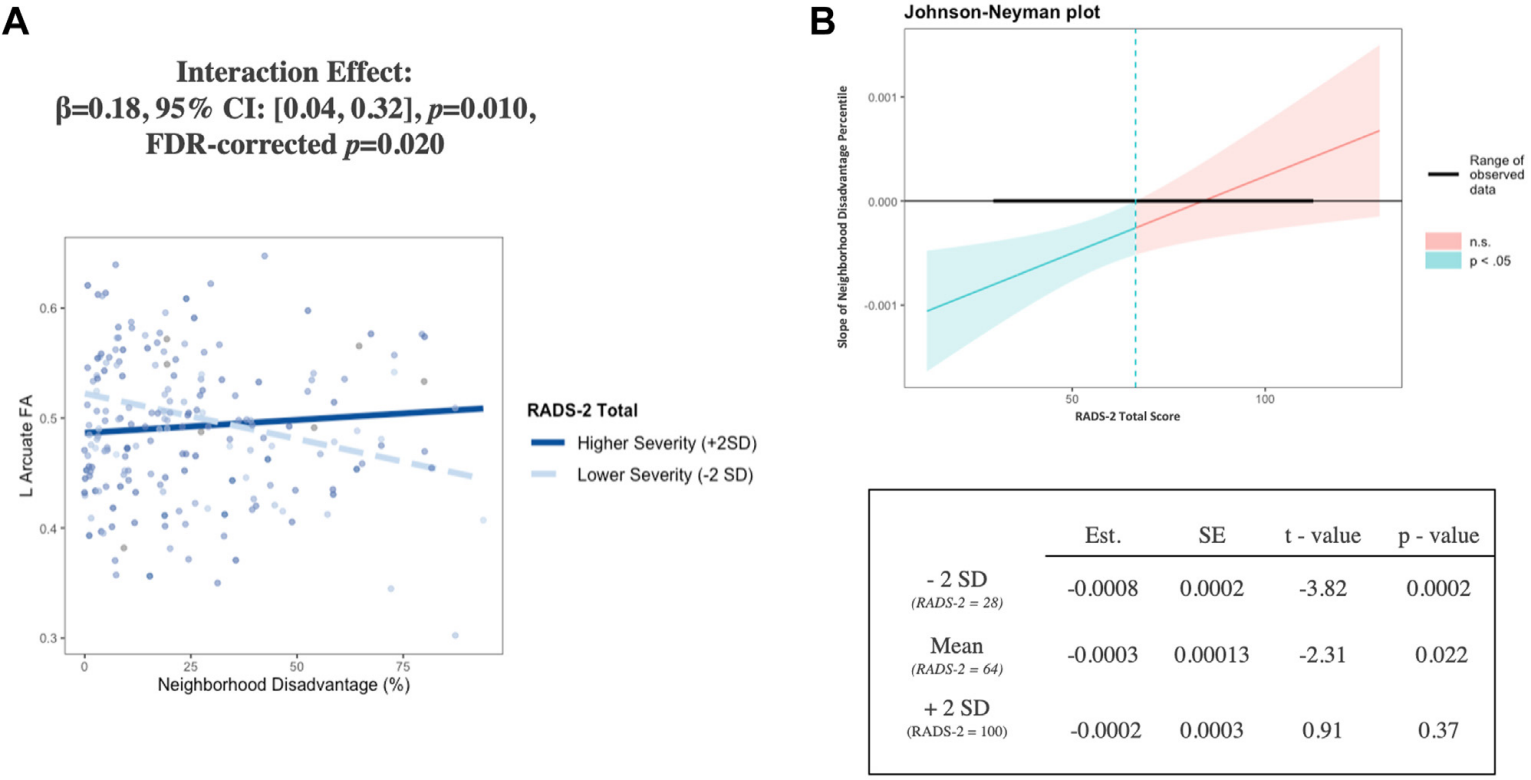
Greater neighborhood disadvantage percentiles were associated with lower fractional anisotropy (FA) of the left (L) arcuate fasciculus in adolescents with lower depression severity, with regression lines visualized at 2 standard deviations above (bolded line) and below (dotted line) mean Reynolds Adolescent Depression Scale (RADS-2) scores **(A)** and with a Johnson-Neyman plot **(B)**. For the scatterplot, all data are displayed without adjustment for covariates for visualization purposes only. For the Johnson-Neyman plot, the inverse association between neighborhood disadvantage percentile and FA of the left arcuate fasciculus was significant only in participants whose depression scores were lower than 66.11 (indicated by the dashed line). The observed range of RADS-2 scores was 30 to 120, as indicated by the bolded black line. A RADS-2 score between 76 and 81 is consistent with mild depression. See the table in **(B)** for simple slopes analysis. See Table 3 for more details. FDR, false discovery rate; n.s., nonsignificant.

**Figure 3:**
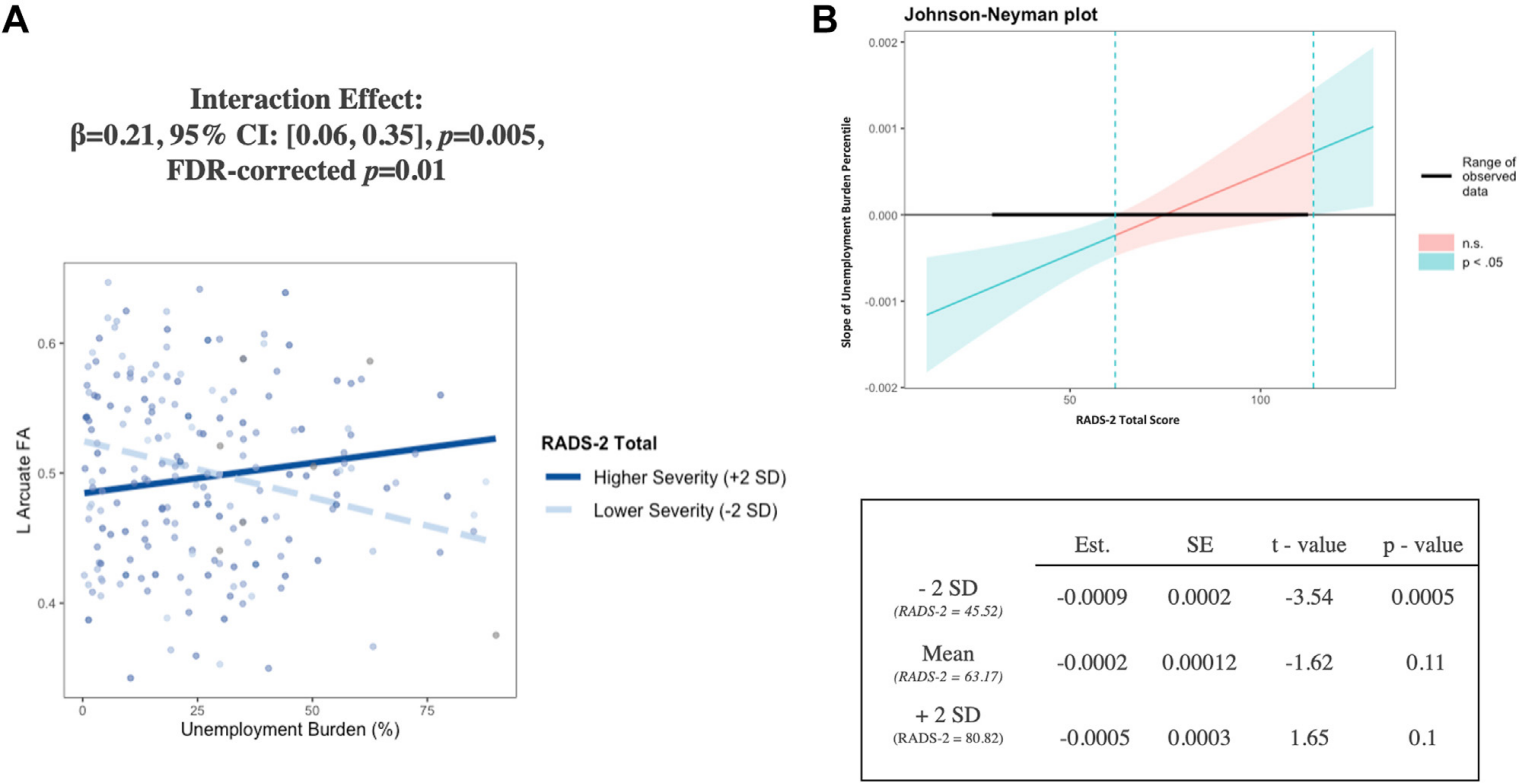
Greater unemployment percentile was associated with lower fractional anisotropy (FA) of the left (L) arcuate fasciculus in adolescents with lower depression severity, with regression lines visualized at 2 standard deviations above (bolded line) and below (dotted line) mean Reynolds Adolescent Depression Scale (RADS-2) scores **(A)** and with a Johnson-Neyman plot **(B)**. For the scatterplot, all data are displayed without adjustment for covariates for visualization purposes only. For the Johnson-Neyman plot, the inverse association between unemployment percentile and FA of the left arcuate fasciculus was significant only in participants whose depression scores were lower than 66.1 and higher than 183.42 (indicated by the dashed lines). However, the highest score possible on the RADS-2 is 120. The observed range of RADS-2 scores was 30 to 120, as indicated by the bolded black line. See the table in **(B)** for simple slopes analysis. See Table 3 for more details. FDR, false discovery rate; n.s., nonsignificant.

In a covariate-adjusted model that included Tanner stage, we found that depression severity significantly moderated the association between neighborhood disadvantage percentile and left arcuate FA (β = 0.18, 95% CI, 0.04 to 0.32, *p* = .010, FDR-corrected *p* = .020). We also found a significant interaction effect between depression severity and unemployment percentile in the left AF (β = 0.21, 95% CI, 0.06 to 0.35, *p* = .005, FDR-corrected *p* = .01) and between depression severity and education disadvantage percentile in the left AF; however, the latter effect did not survive FDR correction (β = 0.15, 95% CI, 0.01 to 0.29, FDR-corrected *p* = .07). When probing these interaction effects, we consistently observed that for adolescents with lower depression severity, higher levels of disadvantage were associated with lower FA in the left AF. However, for adolescents with higher depression severity, there was no significant relationship between socioeconomic disadvantage and FA. See Figure 2, Figure 3 and Table 3. The associations between other socioeconomic indicators and FA in the AF and UF were nonsignificant (all *p*s > .070) and remained nonsignificant without covariate adjustment (see Table S9A, B).

**Table 3:** Summary of Model Results Testing the Interaction Effect of RADS-2 Total Scores and Socioeconomic Disadvantage Percentiles on FA for AF and UF

Tract	Beta Coefficient	SE	95% CI	*t* Value	*p* Value	FDR-Corrected *p* Value	*R*^2^	Δ*R*^2^
Community Disadvantage Percentile								
Left AF FA	0.18	0.07	0.04 to 0.32	2.61	.010^a^	.020^a^	0.224	0.042
Left UF FA	0.04	0.07	−0.10 to 0.18	0.55	.581	.581	0.165	0.002
Right AF FA	0.12	0.08	−0.03 to 0.28	1.57	.118	.236	0.115	0.02
Right UF FA	−0.04	0.07	−0.18 to 0.11	−0.51	.609	.609	0.149	0.002
Education Percentile								
Left AF FA	0.15	0.07	0.01 to 0.29	2.11	.037^a^	.074	0.212	0.03
Left UF FA	0.04	0.08	−0.11 to 0.19	0.54	.590	.590	0.163	0.002
Right AF FA	0.06	0.08	−0.11 to 0.22	0.70	.486	.669	0.106	0.004
Right UF FA	−0.03	0.08	−0.19 to 0.12	−0.43	.669	.669	0.106	0.0012
Unemployment Percentile								
Left AF FA	0.21	0.07	0.06 to 0.35	2.85	.005^b^	.01	0.208	0.049
Left UF FA	0.06	0.08	−0.09 to 0.22	0.84	.400	.400	0.146	0.005
Right AF FA	0.10	0.08	−0.06 to 0.26	1.23	.222	.444	0.079	0.01
Right UF FA	−0.04	0.08	−0.19 to 0.11	−0.52	.603	.603	0.107	0.002

In all linear models, age, sex, body mass index, psychiatric medication use, Tanner stage, study group, race, scan time point, tract length, motion during the scan, and parental education level were included as covariates. FDR-corrected *p* values were calculated based on laterality (left vs. right hemisphere). All reported beta coefficients are standardized.

AF, arcuate fasciculus; FA, fractional anisotropy; FDR, false discovery rate; RADS-2, Reynolds Adolescent Depression Scale; UF, uncinate fasciculus.

a *p* < .05.

b *p* < .01.

As a post hoc analysis, we also tested the indirect effects of neighborhood disadvantage (and any of the indicators) on depression severity via FA in the AF and UF. We found that FA did not significantly mediate the association between neighborhood disadvantage (and the individual indicators) and depression severity (all *p*s > .125).

### Exploratory Analysis: Depression Severity Moderates the Association Between Neighborhood Disadvantage and FA of the Left AF in Girls

As an exploratory analysis, we examined whether sex moderated the association of neighborhood disadvantage (including subsequent individual indicators) with FA in the AF and UF. In a covariate-adjusted model, sex did not significantly moderate the association between FA and neighborhood disadvantage percentile (all *p*s > .063). Similar findings were obtained with subsequent individual indicators (all *p*s > .085).

Given that girls reported higher levels of depressive symptoms than boys in our sample (*p* < .0008), we examined whether the moderating effect of RADS-2 scores on the associations of neighborhood disadvantage with FA in the AF and UF were evident in both sexes. In a covariate-adjusted model that included pubertal stage, depression severity significantly moderated the association between neighborhood disadvantage percentile and left arcuate FA (β = 0.20, 95% CI, 0.03 to 0.37, *p* = .024), poverty percentile (β = 0.18, 95% CI, 0.00 to 0.35, *p* = .047), and unemployment percentile (β = 0.24, 95% CI, 0.05 to 0.44, *p* = .013) only in girls. In boys only, depression severity moderated the association between linguistic isolation and right arcuate FA (β = −0.31, 95% CI, −0.57 to −0.05, *p* = .020). Depression severity did not significantly moderate the association between neighborhood disadvantage for any of the other isolated indicators and FA (all *p*s > .087). When testing the 3-way interaction of depression severity, sex, and disadvantage on FA in the AF and UF, we found no significant effects (all *p*s > .09).

## Discussion

The current study was designed to elucidate the effects of neighborhood-level socioeconomic disadvantage on white matter architecture in the developing adolescent brain. In a sample of 200 youths recruited based on early-life adversity and depression, we found that neighborhood socioeconomic disadvantage, based on data derived from census tracts, was negatively associated with the white matter organization of tracts related to affective and cognitive functioning. Importantly, the effects of neighborhood socioeconomic disadvantage on these white matter tracts were found to be significant over and above the effects of highest level of educational attainment by a parent (i.e., our family-level measure of SES). Interestingly, our results were concentrated in white matter tracts thought to relay information related to language and socioemotional development (i.e., the AF and UF) rather than in tracts typically associated with general cognitive development (e.g., the CC or IFOF). When we examined the individual-level indicators that comprised our composite measure of neighborhood disadvantage, we found that poverty levels and educational attainment explained the observed pattern of associations. In an exploratory analysis, we tested whether depression severity moderated the associations of neighborhood disadvantage with white matter microstructure in the AF and UF. Contrary to our original hypothesis, we found that at higher levels of depression severity, neighborhood disadvantage was not associated with lower white matter organization in these tracts; however, for adolescents with less severe depression, higher levels of neighborhood disadvantage were associated with lower white matter microstructure in the left AF.

Our main findings are consistent with previous research that demonstrated that white matter microstructure is affected by exposure to neighborhood disadvantage in young adults (14). While Bell *et al.* (14) focused specifically on tracts related to emotional processing, our results revealed that the left AF, a tract implicated in language processing and comprehension (33), may be particularly sensitive to the effects of neighborhood-level socioeconomic disadvantage. Moreover, previous research has demonstrated a significant relationship between SES, as measured by parental education level, and FA in the left AF in a normative sample of adolescents (26). These results are consistent with our finding that poverty and educational attainment levels specifically drove our significant higher-level associations of neighborhood disadvantage with FA in the left AF. Importantly, our results extend previous literature by demonstrating that neighborhood contexts influence adolescent white matter over and above effects of family-level SES factors and, furthermore, that the left AF specifically is sensitive to these effects. Because myelination during the adolescent period is an experience-dependent brain maturation process and the dominant form of neuroplasticity that occurs during this period of development, the types of exposures which occur at the neighborhood level are critical for shaping adolescent brain development. Longitudinal studies are necessary to test the precise ways in which environmental exposures interact with mental health state to shape adolescent brain development. Moreover, the extent to which the indices that we identified in our analyses are driven by distinct features of the social environment (e.g., limited resources or opportunities, more unpredictability in day-to-day experiences) and/or more direct exposures to neurotoxicants (e.g., exposure to water contaminants, particulate matter air pollution, endocrine-disrupting chemicals) requires further investigation (5).

We also explored whether depression moderated the effect of neighborhood disadvantage on white matter microstructure in the AF and UF. Because our sample was enriched for depression and depression risk, we used a dimensional measure of depressive severity to test whether the relationship between neighborhood disadvantage and lower FA was stronger in adolescents with higher levels of depression. Interestingly, we obtained results that were contrary to our hypotheses: neighborhood disadvantage was significantly associated with lower FA in adolescents with less severe depressive symptoms but not in adolescents with more severe depressive symptoms. When probing this result further, we found that this significant interaction effect was present only with neighborhood unemployment rates. Interestingly, the authors of a recent study (34) have argued that neighborhood economic (rather than educational) factors more precisely explained brain phenotypes that are linked with adversity—in this case, negative amygdala-prefrontal functional connectivity—which is broadly consistent with our findings. Although speculative, it may be true that in the absence of depression, salient features of neighborhood economic factors have greater opportunity to leave an impact on the developing brain. Nevertheless, in our study, the specificity of unemployment could also be due to the distributions and/or ranges of this variable in our relatively advantaged sample. More research is needed with larger sample sizes, including samples with more participants at the higher end of the neighborhood disadvantage across these different indicators.

Consistent with previous literature (22,23), we also found sex-specific effects in these associations such that influences of neighborhood disadvantage on outcomes of interest were present only in female adolescents. Adolescent depression itself has been found to be associated with lower FA in several of the white matter tracts we examined (19,35) [although see (24,36)]. From the perspective of experience-dependent neuroplasticity, adolescents with depression (and other related conditions) may be less sensitive to environmental influences—for better or for worse—during this period of development. Our results also have important implications for interpreting the studies that have been conducted to date in this area because almost all previous work in this area has been conducted with normative and psychiatrically heathy samples. Thus, it is possible that our understanding of how neighborhood disadvantage affects the brain does not generalize to individuals with clinical symptoms and mental health difficulties. While speculative, one explanation for these results is that adolescents with clinical depression may have experienced rewiring of white matter tracts due to the experiences that contributed to their symptoms and diagnosis (e.g., early adversity) in a manner that renders the system less plastic to environmental influence (21). Under a stress acceleration model (37), premature termination of neuroplasticity may be protective in harsh or unpredictable environmental conditions (although this may come at the cost of maximizing opportunities to learn from positive experiences that scaffold development) (21). That is, depression may impact mechanisms of plasticity in a manner that minimizes openness to environmental influences. However, this hypothesis requires prospective studies that carefully characterize brain development in a high-risk sample of youths before the onset of depression.

Our investigation is not without limitations. First, our study design was observational and cross-sectional. Longitudinal studies are needed to examine whether major changes in neighborhood disadvantage correspond to changes in microstructure in the tracts we have identified. Second, the way we measured neighborhood disadvantage is limited by our lack of extensive residential address history and a reliance on census tracts provided by the CalEnviroScreen, which may be too broad, may not capture the same time periods for all indicators, and may not accurately represent neighborhood boundaries (38,39). Thus, research that uses more standard socioeconomic indicators (16,40) in combination with prospective data is needed to comprehensively track and measure the timing of neighborhood- and area-level exposures and how that affects the developing brain (13,41). There is also the fact that socioeconomic factors often co-occur (e.g., poverty and educational attainment), with interrelated or compounding effects on the developing brain; therefore, caution is needed in interpreting these results in the absence of samples recruited for and evaluated specifically based on their exposures to one factor but not the other. Third, regarding the generalizability of our findings, our sample was recruited from an advantaged community, and therefore disadvantage in this context may not reflect what is seen elsewhere. Fourth, our tractography methods are limited in resolving areas with crossing fibers (42) and may generate invalid bundles (43). Despite these limitations, diffusion-weighted imaging is currently the only tool to map short- and long-range white matter connectivity pathways in the living brain; advances in tractography methods, particularly in regions with more anatomical complexity, are needed to improve our ability to understand environmental effects on white matter microstructure (43).

Additionally, we did not obtain parental history of depression in both samples (this information was collected only in the TIGER study), making adequate statistical control of heritable liabilities in socioemotional functioning, such as a family history of psychopathology, more challenging. Finally, it is important to consider more precise definitions of a “neighborhood.” Adolescents’ daily exposures to various psychosocial inputs can, and often do, extend beyond the census definition of a neighborhood. For example, exposure to favorable school environments, which may or may not fall within a child’s immediate neighborhood, have been associated with greater connectivity of the auditory and retrosplenial temporal network and higher-order cognitive networks but not with white matter connectivity (44). Geolocation technology has also made it possible to track mobility patterns of adolescents (45); this information, combined with census tract data or other sources of environmental information, could be used to richly characterize adolescent exposures outside the home and elucidate their relationships with brain development.

### Conclusions

In sum, our findings underscore the importance of considering neighborhood-level factors when examining the effects of socioeconomic disadvantage on the brain. Prospective studies that examine these questions using a clinical trial design, including recent work on infant development using cash aid for families experiencing poverty (46), are needed to determine whether such interventions will influence the patterns of white matter microstructure that we report in this study. Overall, our results suggest that public health policies that are aimed at improving conditions at the neighborhood and community levels are likely to lead to greater gains in neurobiological and psychosocial outcomes among children and adolescents.

## Supplementary Material

Supplement

Key Resources Table

## References

[1] Holstein B.E., Currie C., Boyce W., Damsgaard M.T., Gobina I., Kökönyei G. (2009). Socio-economic inequality in multiple health complaints among adolescents: International comparative study in 37 countries. Int J Public Health.

[2] Reiss F. (2013). Socioeconomic inequalities and mental health problems in children and adolescents: A systematic review. Soc Sci Med.

[3] Farah M.J. (2018). Socioeconomic status and the brain: Prospects for neuroscience-informed policy. Nat Rev Neurosci.

[4] Rakesh D., Whittle S. (2021). Socioeconomic status and the developing brain – A systematic review of neuroimaging findings in youth. Neurosci Biobehav Rev.

[5] Cardenas-Iniguez C., Burnor E., Herting M.M. (2022). Neurotoxicants, the developing brain, and mental health. Biol Psychiatry Glob Open Sci.

[6] Noble K.G., Houston S.M., Brito N.H., Bartsch H., Kan E., Kuperman J.M. (2015). Family income, parental education and brain structure in children and adolescents. Nat Neurosci.

[7] Noble K.G., Giebler M.A. (2020). The neuroscience of socioeconomic inequality. Curr Opin Behav Sci.

[8] Evans G.W. (2004). The environment of childhood poverty. Am Psychol.

[9] Leventhal T., Brooks-Gunn J. (2000). The neighborhoods they live in: The effects of neighborhood residence on child and adolescent outcomes. Psychol Bull.

[10] Hyde L.W., Gard A.M., Tomlinson R.C., Burt S.A., Mitchell C., Monk C.S. (2020). An ecological approach to understanding the developing brain: Examples linking poverty, parenting, neighborhoods, and the brain. Am Psychol.

[11] Miller J.G., López V., Buthmann J.L., Garcia J.M., Gotlib I.H. (2022). A social gradient of cortical thickness in adolescence: Relationships with neighborhood socioeconomic disadvantage, family socioeconomic status, and depressive symptoms. Biol Psychiatry Glob Open Sci.

[12] Tooley U.A., Mackey A.P., Ciric R., Ruparel K., Moore T.M., Gur R.C. (2020). Associations between neighborhood SES and functional brain network development. Cereb Cortex.

[13] Ho T.C., King L.S. (2021). Mechanisms of neuroplasticity linking early adversity to depression: Developmental considerations. Transl Psychiatry.

[14] Bell K.L., Purcell J.B., Harnett N.G., Goodman A.M., Mrug S., Schuster M.A. (2021). White matter microstructure in the young adult brain varies with neighborhood disadvantage in adolescence. Neuroscience.

[15] Fuhrmann D., Knoll L.J., Blakemore S.J. (2015). Adolescence as a sensitive period of brain development. Trends Cogn Sci.

[16] Gard A.M., Maxwell A.M., Shaw D.S., Mitchell C., Brooks-Gunn J., McLanahan S.S. (2021). Beyond family-level adversities: Exploring the developmental timing of neighborhood disadvantage effects on the brain. Dev Sci.

[17] Reiss F., Meyrose A.K., Otto C., Lampert T., Klasen F., Ravens-Sieberer U. (2019). Socioeconomic status, stressful life situations and mental health problems in children and adolescents: Results of the German BELLA cohort-study. PLoS One.

[18] Whittle S., Lichter R., Dennison M., Vijayakumar N., Schwartz O., Byrne M.L. (2014). Structural brain development and depression onset during adolescence: A prospective longitudinal study. Am J Psychiatry.

[19] LeWinn K.Z., Connolly C.G., Wu J., Drahos M., Hoeft F., Ho T.C. (2014). White matter correlates of adolescent depression: Structural evidence for frontolimbic disconnectivity. J Am Acad Child Adolesc Psychiatry.

[20] Connolly C.G., Ho T.C., Blom E.H., LeWinn K.Z., Sacchet M.D., Tymofiyeva O. (2017). Resting-state functional connectivity of the amygdala and longitudinal changes in depression severity in adolescent depression. J Affect Disord.

[21] Ho T.C. (2019). Stress and neurodevelopment in adolescent depression. Biol Psychiatry.

[22] Leventhal T., Brooks-Gunn J. (2003). Moving to opportunity: An experimental study of neighborhood effects on mental health. Am J Public Health.

[23] King C., Huang X., Dewan N.A. (2022). Continuity and change in neighborhood disadvantage and adolescent depression and anxiety. Health Place.

[24] Ho T.C., Sisk L.M., Kulla A., Teresi G.I., Hansen M.M., Wu H., Gotlib I.H. (2021). Sex differences in myelin content of white matter tracts in adolescents with depression. Neuropsychopharmacology.

[25] Dufford A.J., Evans G.W., Dmitrieva J., Swain J.E., Liberzon I., Kim P. (2020). Prospective associations, longitudinal patterns of childhood socioeconomic status, and white matter organization in adulthood. Hum Brain Mapp.

[26] Vanderauwera J., van Setten E.R.H., Maurits N.M., Maassen B.A.M. (2019). The interplay of socio-economic status represented by paternal educational level, white matter structure and reading. PLoS One.

[27] Walker J.C., Teresi G.I., Weisenburger R.L., Segarra J.R., Ojha A., Kulla A. (2020). Study protocol for teen inflammation glutamate emotion research (TIGER). Front Hum Neurosci.

[28] King L.S., Humphreys K.L., Camacho M.C., Gotlib I.H. (2019). A person-centered approach to the assessment of early life stress: Associations with the volume of stress-sensitive brain regions in early adolescence. Dev Psychopathol.

[29] Chahal R., Miller J.G., Yuan J.P., Buthmann J.L., Gotlib I.H. (2022). An exploration of dimensions of early adversity and the development of functional brain network connectivity during adolescence: Implications for trajectories of internalizing symptoms. Dev Psychopathol.

[30] Yeatman J.D., Dougherty R.F., Myall N.J., Wandell B.A., Feldman H.M. (2012). Tract profiles of white matter properties: Automating fiber-tract quantification. PLoS One.

[31] Kircanski K., Sisk L.M., Ho T.C., Humphreys K.L., King L.S., Colich N.L. (2019). Early life stress, cortisol, frontolimbic connectivity, and depressive symptoms during puberty. Dev Psychopathol.

[32] Reynolds W.M. (2002).

[33] Ivanova M.V., Zhong A., Turken A., Baldo J.V., Dronkers N.F. (2021). Functional contributions of the arcuate fasciculus to language processing. Front Hum Neurosci.

[34] Ramphal B., DeSerisy M., Pagliaccio D., Raffanello E., Rauh V., Tau G. (2020). Associations between amygdala-prefrontal functional connectivity and age depend on neighborhood socioeconomic status. Cereb Cortex Commun.

[35] Xu E.P., Nguyen L., Leibenluft E., Stange J.P., Linke J.O. (2023). A meta-analysis on the uncinate fasciculus in depression. Psychol Med.

[36] Van Velzen L.S., Kelly S., Isaev D., Aleman A., Aftanas L.I., Bauer J. (2020). White matter disturbances in major depressive disorder: A coordinated analysis across 20 international cohorts in the ENIGMA MDD working group. Mol Psychiatry.

[37] Callaghan B.L., Tottenham N. (2016). The stress acceleration hypothesis: Effects of early-life adversity on emotion circuits and behavior. Curr Opin Behav Sci.

[38] Browning C.R., Soller B., Jackson A.L. (2015). Neighborhoods and adolescent health-risk behavior: An ecological network approach. Soc Sci Med.

[39] Coulton C.J., Korbin J., Chan T., Su M. (2001). Mapping residents’ perceptions of neighborhood boundaries: A methodological note. Am J Community Psychol.

[40] Taylor R.L., Cooper S.R., Jackson J.J., Barch D.M. (2020). Assessment of neighborhood poverty, cognitive function, and prefrontal and hippocampal volumes in children. JAMA Netw Open.

[41] Gabard-Durnam L.J., McLaughlin K.A. (2019). Do sensitive periods exist for exposure to adversity?. Biol Psychiatry.

[42] Behrens T.E., Berg H.J., Jbabdi S., Rushworth M.F., Woolrich M.W. (2007). Probabilistic diffusion tractography with multiple fibre orientations: What can we gain?. Neuroimage.

[43] Maier-Hein K.H., Neher P.F., Houde J.C., Côté M.A., Garyfallidis E., Zhong J. (2017). The challenge of mapping the human connectome based on diffusion tractography. Nat Commun.

[44] Rakesh D., Zalesky A., Whittle S. (2023). The role of school environment in brain structure, connectivity, and mental health in children: A multimodal investigation. Biol Psychiatry Cogn Neurosci Neuroimaging.

[45] Saragosa-Harris N.M., Cohen A.O., Reneau T.R., Villano W.J., Heller A.S., Hartley C.A. (2022). Real-world exploration increases across adolescence and relates to affect, risk taking, and social connectivity. Psychol Sci.

[46] Troller-Renfree S.V., Costanzo M.A., Duncan G.J., Magnuson K., Gennetian L.A., Yoshikawa H. (2022). The impact of a poverty reduction intervention on infant brain activity. Proc Natl Acad Sci USA.

