# Supplementary material for: Neighborhood Socioeconomic Disadvantage and White Matter Microstructure of the Arcuate Fasciculus and Uncinate Fasciculus in Adolescents
Source: Biol Psychiatry Glob Open Sci. 2023 Oct 14;4(1):61–72. doi: 10.1016/j.bpsgos.2023.10.002 (PMC10709004; doi:10.1016/j.bpsgos.2023.10.002)
Supplement: Supplement [file mmc1.pdf]

SUPPLEMENTARY INFORMATION

Neighborhood Socioeconomic Disadvantage and White Matter Microstructure of the Arcuate Fasciculus and Uncinate Fasciculus in Adolescents

Kulla *et al.*

Publication Overlap

A subset of the sample (*N*=83 and *N*=75) had microstructural metrics from the uncinate fasciculus published previously (1,2). The current study focuses on a significantly larger sample (*N*=200) and on relating white matter microstructure of the arcuate fasciculus with census tract data, both of which are novel data sources that have not been previously published by our group.

Methods

Participants

Data from the present study were collected through two ongoing longitudinal neuroimaging studies at Stanford University: The Teen Inflammation Glutamate Emotion Research (TIGER) study ((3); NIH grant: K01MH117442) and the Early Life Stress (ELS) study (NIH grant: R37MH101495). Participants from both study cohorts were recruited from the San Francisco Bay Area using internal referral systems, flyering at local venues, and social media advertisements. Data from both cohorts were collected between 2017-2021. Because the primary goal of the TIGER study was to compare depressed and nondepressed adolescents, inclusion/exclusion criteria differed for these groups. Inclusion criteria for all TIGER participants at the baseline assessment included being ages 13–18 years, endorsing Tanner stage greater than or equal to 3, and fluency in English. Depressed adolescents also had to meet DSM-IV diagnostic criteria for a depressive disorder, assessed using the Kiddie Schedule for Affective Disorders and Schizophrenia, Present and Lifetime (K-SADS-PL) or, if subthreshold on the K-SADS-PL, a total *t*-score > 55 on the Children's Depression Rating Scale-Revised (4). Exclusion criteria included a current or lifetime criteria for psychosis, mania, or substance dependence (see below for additional exclusion criteria). Healthy control (CTL) participants for TIGER had the same inclusion and exclusion criteria with the exception that they could not meet criteria for current or lifetime diagnoses for any Axis I disorder and could not have a first-degree relative with a history of suicide or suspected history of depression, psychosis/mania, or substance dependence (see Walker et al. (3) for more details).

Participants for ELS were recruited as part of a four-wave longitudinal study characterizing the effects of early life stress on brain development across the pubertal transition (5, 6). Inclusion criteria at the baseline assessment for ELS included English fluency, being between the ages of 9-12 years, and being early in pubertal development (i.e., menarche was exclusionary for girls). More details about the types of adverse experiences to which participants in the ELS study were exposed are presented in Table 2 of King et al. (5), with witnessing severe illness (69%), moving (63%), experiencing family verbal fights and arguments, experiencing severe illness (50%), the death of someone close (50%), separation of someone close (50%), or family mental illness/substance use (46%) being the most common. Adolescents were racially and ethnically diverse (37% self-reported as White/Caucasian), and 31% qualified as low income based on their income-to-needs ratios (5). In the present investigation, we included data from the third wave of the ELS study, when participants were ages 14–17 years, because the ages and pubertal stages of the ELS participants at this time point were comparable to those of the adolescents participating in the TIGER study.

Additional exclusion criteria for both studies were the same: history of neurological disorder or major medical illness, any cognitive or physical challenges that would limit adolescents' ability to understand or complete study procedures, contraindications for MRI (e.g., braces, claustrophobia), and symptoms of concussion within the past 6 weeks or loss of consciousness during any lifetime concussion. In accordance with the Declaration of Helsinki, all participants provided informed assent and their parent(s)/legal guardian(s) provided informed consent. All participants were compensated for study participation with gift cards. TIGER was approved by the Institutional Review Boards at the University of California, San Francisco and Stanford University and ELS was approved by the Institutional Review Board at Stanford University.

Neuroimaging Acquisition

For ELS participants, a high-resolution T1-weighted anatomical scan was acquired using an SPGR sequence (TR/TE/TI = 6.24/2.34/450 ms; flip angle=12°; 186 sagittal slices; 0.9 mm isotropic voxels). For TIGER participants, a high-resolution T1-weighted anatomical scan was acquired using an SPGR sequence (TR/TE/TI = 8.2/3.2/600 ms; flip angle=12°; 156 axial slices; 1.0 mm isotropic voxels). In both studies, a diffusion-weighted imaging scan was acquired using an EPI sequence (TR/TE = 8500/93.5 ms; 64 axial slices; 2 mm isotropic voxels; 60 b=2000 diffusion-weighted directions, and 6 b=0 acquisitions at the beginning of the scan; anterior/posterior phase encoding direction).

Deterministic Tractography

Diffusion MRI data were processed using the open source mrVista software distribution developed by the VISTA lab (https://vistalab.stanford.edu/). Whole-brain fiber tracts were first mapped onto an anterior-posterior commissure-aligned T1-weighted image based on a deterministic algorithm (7, 8). As in previous work (8, 9), we minimized the effects of motion on the data by first excluding directions (i.e., volumes) where relative motion in the translational directions or the rotational directions exceeded 5 mm or 1.5°, respectively. Streamlines in each of the tracts of interest–bilateral arcuate fasciculus (AF), cingulum cingulate (CC), corticospinal tract (CST), inferior fronto-occipital fasciculus (IFOF), and uncinate fasciculus (UF) –were automatically generated using a two planar waypoint region of interest (ROI) approach and then seeing these two ROIs in accordance with well-established probabilistic fiber groupings (see Yeatman et al. (7) for more details). Candidate fibers were then assessed based on similarity to the standard probability map (10, 11). We excluded outliers, defined as exceeding 4 standard deviations away from the spatial core of the tract, until no outlier volumes existed in the tract (1, 8). All tracts were visually inspected by the first and senior author for consistency. As AFQ computes diffusivity metrics for 100 evenly spaced nodes along the tract, we averaged diffusivity metrics along the entire tract for a more reliable estimate, as in our previous work (1, 12).

Neighborhood Disadvantage Data (CalEnviroScreen 3.0)

The socioeconomic indicators constituted the following: educational attainment, poverty, housing burden, linguistic isolation, and unemployment. Educational attainment percentiles were measured as the percent of the population older than age 25 with less than a high-school education, and poverty percentiles were measured by the percent of the population living below two times the federal poverty level. Both measures were 5-year estimates between 2011-2015. Housing burden percentiles were calculated as the percent of households that are both low income (family income <80% of the Housing and Urban Development Area Median Family Income) and significantly burdened by housing costs over a 5-year estimate (2009-2013). Linguistic isolation was defined as the percentage of limited English-speaking households (2011-2015). Unemployment was defined as the percent of the population over the age of 16 that is unemployed and eligible for the labor force (excluding retirees, students, homemakers, institutionalized persons except prisoners, those not looking for work, and military personnel on active duty) over a 5-year estimate (2011-2015).

Results Using CalEnviroScreen 4.0

Higher Percentiles of Neighborhood Disadvantage are Associated with Lower Fractional Anisotropy in Arcuate Fasciculus and Uncinate Fasciculus

Higher percentiles of neighborhood disadvantage were significantly associated with lower FA in the right UF only after applying FDR-correction (β=-0.27, 95% CI: [-0.44, -0.10], FDR-corrected p=0.01). See **Tables S2CD** for more details.

Post-Hoc Analysis: Depression Severity Moderates the Association Between Neighborhood Disadvantage and Fractional Anisotropy of the Left Arcuate Fasciculus

Depression severity significantly moderated the association between neighborhood disadvantage percentile and left arcuate FA (β=0.18, 95% CI: [0.04, 0.32], FDR-corrected p=0.022). Depression severity also significantly moderated the association between education disadvantage percentile and FA in left arcuate, but this effect did not survive FDR correction (β=0.15, 95% CI: [0.02, 0.29], p=0.030). See **Table S9C** for more details.

Exploratory Analysis: Depression Severity Moderates the Association Between Neighborhood Disadvantage and Fractional Anisotropy of the Left Arcuate Fasciculus in Girls

We found that depression severity moderated the association between neighborhood disadvantage percentile and left arcuate FA (β=0.18, 95% CI: [0.00, 0.35], p=0.045). Depression severity did not significantly moderate the association between the other indices and FA in girls (all ps>0.094). In boys, no significant effects were found (all ps> 0.052). Finally, when testing the three-way interaction of depression severity, sex, and disadvantage, no significant effects were found (ps>0.103).

Figure S1. Histogram of Tanner Score.

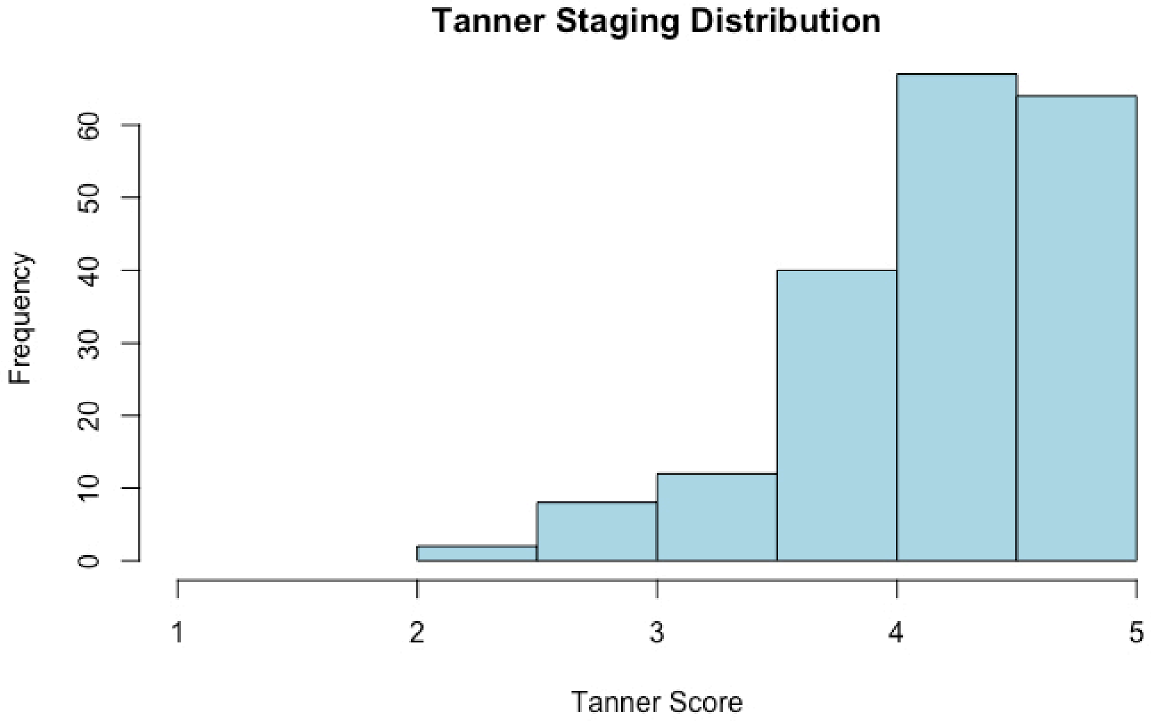

Figure S2. Density plots visualizing the distribution of neighborhood disadvantage and subsequent neighborhood indicators. All data are displayed without adjustment covariates for visualization only.

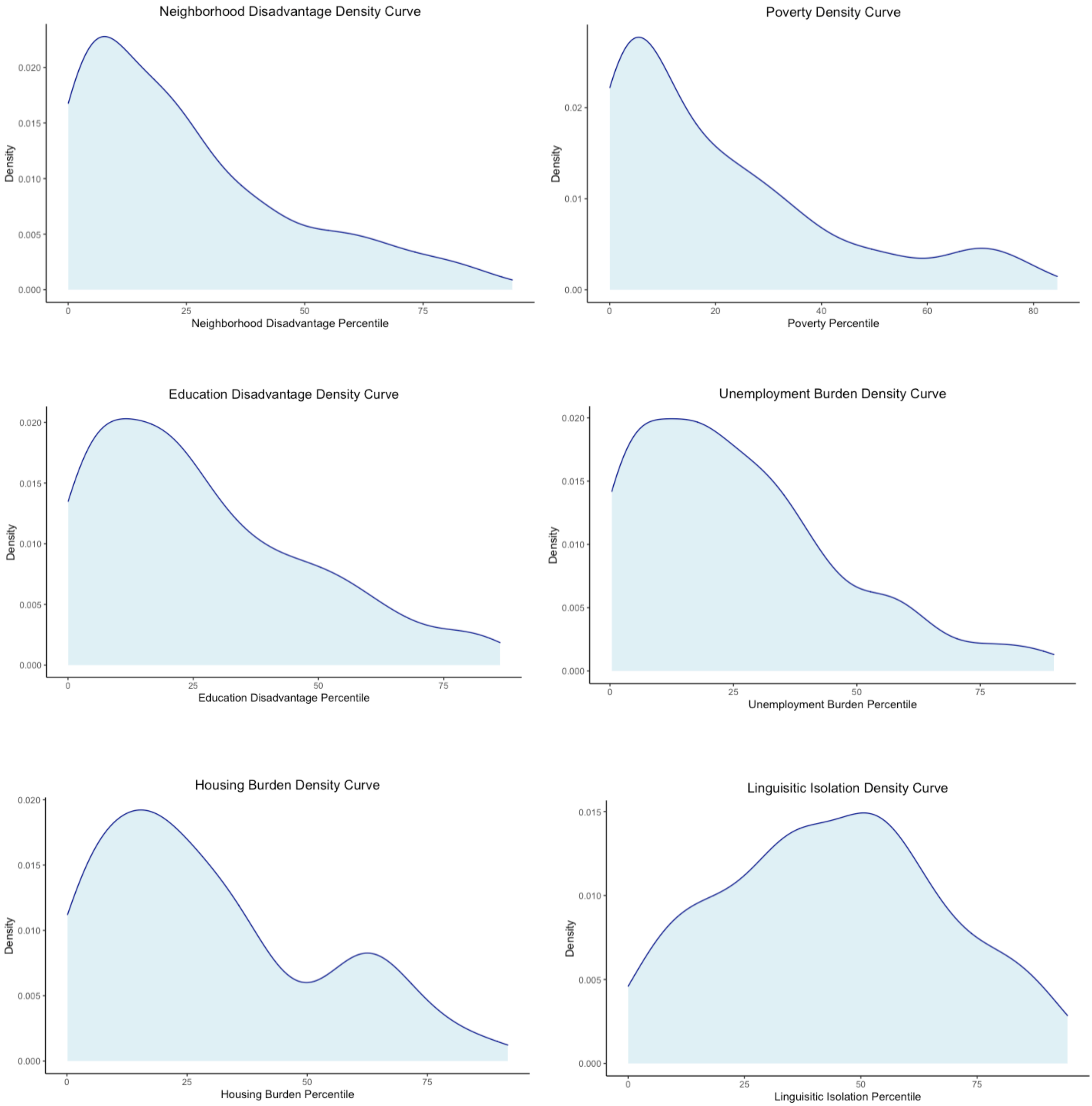

Figure S3A. Correlation matrix to visualize the associations between key predictors and outcomes. Only correlations significant at the $p = 0.05$ level are visualized. Positive associations are depicted in warmer colors and negative associations are depicted in cooler colors.

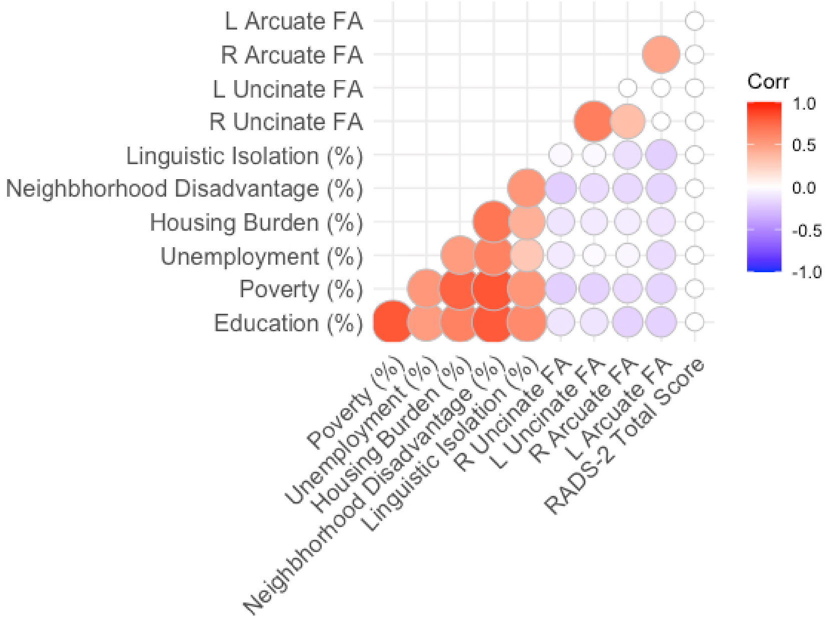

Figure S3B. Correlation matrix to visualize association between parental level education (family SES) and neighborhood disadvantage. Positive associations are depicted in warmer colors and negative associations are depicted in cooler colors.

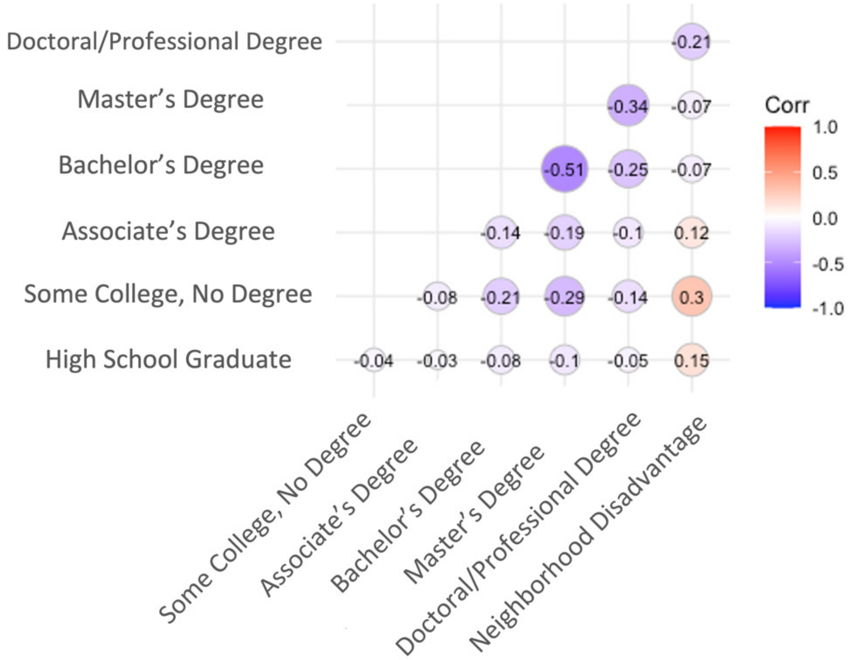

Table S1. Descriptive statistics for demographic and primary variables of interest in the full sample and by study. Significance values in the final column indicate whether there are study group differences. χ^2 tests were used to examine group differences in categorical variables and Student's *t*-tests (assuming homogeneity of variance) were used to examine group differences in continuous variables. Motion refers to the average amount of movement across six dimensions during the diffusion MRI scan, where negative values refer to displacement in the leftward direction for x, the posterior direction for y, the inferior direction for z, leftward tilt for pitch, counterclockwise rotation for roll, and downward tilt for yaw. BMI=body mass index; FA=fractional anisotropy; SD=standard deviation.

	**ELS (*N*=122)**	**TIGER (*N*=78)**	**Total (*N*=200)**	***p*-value**
**Age**				0.104
Mean (SD)	15.809 (1.298)	16.113 (1.263)	15.927 (1.290)	
Range	13.065 - 20.080	13.150 - 18.230	13.065 - 20.080	
**Sex**				0.356
Male	55 (45.1%)	30 (38.5%)	85 (42.5%)	
Female	67 (54.9%)	48 (61.5%)	115 (57.5%)	
**Gender**				0.767
Male	53 (43.4%)	30 (38.5%)	83 (41.5%)	
Female	64 (52.5%)	45 (57.7%)	109 (54.5%)	
Non-binary	5 (4.1%)	3 (3.8%)	8 (4.0%)	
**Ethnicity**				0.198
Hispanic or Latino	14 (11.5%)	14 (17.9%)	28 (14.0%)	
Non-Hispanic or Latino	108 (88.5%)	64 (82.1%)	172 (86.0%)	
**Race**				0.545
American Indian or Alaska Native	3 (2.5%)	2 (2.6%)	5 (2.5%)	
Asian	19 (15.6%)	17 (21.8%)	36 (18.0%)	
Black or African American	10 (8.2%)	2 (2.6%)	12 (6.0%)	
Native Hawaiian or Other PI	0 (0.0%)	0 (0.0%)	0 (0.0%)	
White	59 (48.4%)	36 (46.2%)	95 (47.5%)	
Multiracial	18 (14.8%)	14 (17.9%)	32 (16.0%)	
Other	13 (10.7%)	7 (9.0%)	20 (10.0%)	
**Scanned during COVID-19**				0.001
No	84 (68.9%)	69 (88.5%)	153 (76.5%)	
Yes	38 (31.1%)	9 (11.5%)	47 (23.5%)	
**Tanner Score**				0.884
# missing	0	7	7	

Mean (SD)	4.410 (0.600)	4.423 (0.552)	4.415 (0.581)	
Range	2.000 - 5.000	3.000 - 5.000	2.000 - 5.000	
**Parental Level of Education**				0.144
# missing	2	4	6	
Less than a high school diploma	0 (0%)	0 (0%)	0 (0%)	
High School graduate or equivalent (GED)	2 (1.7%)	1 (1.4%)	3 (1.5%)	
Some college, no degree	15 (12.5%)	6 (8.1%)	21 (10.8%)	
Associate degree (e g AA, AS)	9 (7.5%)	1 (1.4%)	10 (5.2%)	
Bachelor's degree (e g BA, BS)	34 (28.3%)	18 (24.3%)	52 (26.8%)	
Master's degree (e g MA, MS, MEd)	47 (39.2%)	32 (43.2%)	79 (40.7%)	
Doctoral or Professional degree (MD, DDS, DVM, PhD, EdD)	13 (10.8%)	16 (21.6%)	29 (14.9%)	
**Psychiatric Medication Status**				< 0.001
No Medication Use	112 (91.8%)	50 (64.1%)	162 (81.0%)	
Medication Use	10 (8.2%)	28 (35.9%)	38 (19.0%)	
**Corticosteroid Use**				0.34
# missing	8	0	8	
No Corticosteroid Use	104 (91.2%)	74 (94.9%)	178 (92.7%)	
Corticosteroid Use	10 (8.8%)	4 (5.1%)	14 (7.3%)	
**BMI**				0.136
# missing	0	1	1	
Mean (SD)	21.717 (4.533)	22.731 (4.832)	22.109 (4.665)	
Range	15.130 - 39.247	14.747 - 38.514	14.747 - 39.247	
**Diagnostic History of Major Depressive Disorder**				< 0.001
No	90 (73.8%)	21 (26.9%)	111 (55.5%)	
Yes	32 (26.2%)	57 (73.1%)	89 (44.5%)	
**RADS-2 Dysphoric Mood Score**				< 0.001
# missing	5	0	5	
Mean (SD)	18.188 (5.097)	22.141 (6.183)	19.769 (5.872)	
Range	8.000 - 27.000	8.000 - 32.000	8.000 - 32.000	
**RADS-2 Anhedonia/Negative Affect Score**				< 0.001
# missing	5	0	5	

Mean (SD)	11.085 (3.282)	13.795 (4.238)	12.169 (3.916)	
Range	7.000 - 25.000	7.000 - 25.000	7.000 - 25.000	
**RADS-2 Negative Self-Evaluation Score**				< 0.001
# missing	7	0	7	
Mean (SD)	13.426 (4.726)	17.218 (6.428)	14.959 (5.772)	
Range	8.000 - 29.000	8.000 - 29.000	8.000 - 29.000	
**RADS-2 Somatic Complaints Score**				0.036
# missing	5	0	5	
Mean (SD)	16.701 (4.276)	18.090 (4.814)	17.256 (4.538)	
Range	7.000 - 24.000	7.000 - 28.000	7.000 - 28.000	
**RADS-2 Total Score**				< 0.001
# missing	7	0	7	
Mean (SD)	59.313 (14.938)	71.244 (19.258)	64.135 (17.767)	
Range	30.000 - 101.000	31.000 - 112.000	30.000 - 112.000	
**Education Percentile Score**				0.008
# missing	1	2	3	
Mean (SD)	30.229 (22.346)	21.872 (19.605)	27.005 (21.666)	
Range	0.180 - 84.570	0.040 - 86.270	0.040 - 86.270	
**Poverty Percentile Score**				0.015
Mean (SD)	24.489 (22.596)	16.821 (19.695)	21.499 (21.785)	
Range	0.030 - 84.500	0.100 - 80.010	0.030 - 84.500	
**Unemployment Percentile Score**				0.021
Mean (SD)	28.937 (22.364)	21.986 (17.501)	26.226 (20.837)	
Range	0.360 - 89.910	0.770 - 85.160	0.360 - 89.910	
**Housing Burden Percentile Score**				0.023
# missing	1	0	1	
Mean (SD)	33.165 (24.122)	25.547 (20.985)	30.179 (23.190)	
Range	0.130 - 91.700	0.930 - 91.060	0.130 - 91.700	
**Linguistic Isolation Percentile Score**				0.107
# missing	2	0	2	

Mean (SD)	46.320 (23.365)	40.725 (24.385)	44.116 (23.868)	
Range	0.000 - 93.350	0.000 - 94.410	0.000 - 94.410	
**Population Characteristics Percentile Score**				< 0.001
# missing	1	0	1	
Mean (SD)	29.779 (23.277)	17.225 (19.058)	24.858 (22.525)	
Range	0.710 - 93.870	0.030 - 72.950	0.030 - 93.870	
**Pollution Burden Percentile Score**				0.758
Mean (SD)	32.239 (20.717)	33.123 (18.305)	32.584 (19.769)	
Range	0.290 - 83.870	1.410 - 79.510	0.290 - 83.870	
**Left Arcuate FA mean**				0.702
# missing	2	0	2	
Mean (SD)	0.492 (0.032)	0.494 (0.031)	0.493 (0.032)	
Range	0.384 - 0.569	0.422 - 0.584	0.384 - 0.584	
**Left Arcuate AD mean**				0.141
# missing	2	0	2	
Mean (SD)	0.948 (0.035)	0.956 (0.044)	0.951 (0.039)	
Range	0.819 - 1.025	0.860 - 1.094	0.819 - 1.094	
**Left Arcuate MD mean**				0.089
# missing	2	0	2	
Mean (SD)	0.595 (0.019)	0.600 (0.020)	0.597 (0.020)	
Range	0.550 - 0.655	0.547 - 0.654	0.547 - 0.655	
**Left Arcuate RD mean**				0.363
# missing	2	0	2	
Mean (SD)	0.419 (0.025)	0.422 (0.023)	0.420 (0.024)	
Range	0.354 - 0.504	0.357 - 0.475	0.354 - 0.504	
**Left Arcuate Tractlength (mm)**				0.555
Mean (SD)	11552.970 (44200.475)	15743.707 (55520.960)	13187.357 (48835.153)	
Range	866.110 – 301000.0	911.120 – 330000.0	866.110 – 330000.0	
**Right Arcuate FA mean**				0.572

# missing	17	7	24	
Mean (SD)	0.466 (0.031)	0.469 (0.037)	0.468 (0.034)	
Range	0.390 - 0.528	0.363 - 0.554	0.363 - 0.554	
**Right Arcuate AD mean**				0.081
# missing	17	7	24	
Mean (SD)	0.928 (0.035)	0.939 (0.047)	0.933 (0.040)	
Range	0.841 - 1.005	0.833 - 1.082	0.833 - 1.082	
**Right Arcuate MD mean**				0.073
# missing	17	7	24	
Mean (SD)	0.594 (0.018)	0.600 (0.019)	0.597 (0.019)	
Range	0.550 - 0.659	0.561 - 0.640	0.550 - 0.659	
**Right Arcuate RD mean**				0.441
# missing	17	8	25	
Mean (SD)	0.428 (0.022)	0.430 (0.022)	0.429 (0.022)	
Range	0.377 - 0.487	0.373 - 0.508	0.373 - 0.508	
**Right Arcuate Tractlength (mm)**				0.311
# missing	1	1	2	
Mean (SD)	4212.576 (11267.74)	2858.182 (3937.88)	3685.867 (9151.93)	
Range	631.290 - 94949.0	705.780 - 31488.0	631.290 - 94949.0	
**Left UF FA mean**				0.22
# missing	1	2	3	
Mean (SD)	0.434 (0.030)	0.440 (0.034)	0.437 (0.032)	
Range	0.354 - 0.538	0.348 - 0.525	0.348 - 0.538	
**Left UF AD mean**				0.207
# missing	1	2	3	
Mean (SD)	1.084 (0.034)	1.091 (0.035)	1.087 (0.034)	
Range	0.979 - 1.163	1.006 - 1.184	0.979 - 1.184	
**Left UF MD mean**				0.830
# missing	1	2	3	
Mean (SD)	0.712 (0.018)	0.712 (0.023)	0.712 (0.020)	
Range	0.665 - 0.754	0.658 - 0.764	0.658 - 0.764	

**Left UF RD mean**				0.588
# missing	1	2	3	
Mean (SD)	0.526 (0.025)	0.523 (0.031)	0.525 (0.028)	
Range	0.447 - 0.587	0.456 - 0.607	0.447 - 0.607	
**Left UF Tractlength (mm)**				0.467
# missing	0	1	1	
Mean (SD)	3959.298 (2518.8)	3713.914 (1948.3)	3864.351 (2312.7)	
Range	1167.70 - 16480.0	1261.500 - 10542.0	1167.70 - 16480.0	
**Right UF FA mean**				0.929
Mean (SD)	0.433 (0.029)	0.433 (0.029)	0.433 (0.029)	
Range	0.351 - 0.502	0.340 - 0.493	0.340 - 0.502	
**Right UF AD mean**				0.918
Mean (SD)	1.093 (0.033)	1.093 (0.029)	1.093 (0.031)	
Range	1.012 - 1.191	1.007 - 1.149	1.007 - 1.191	
**Right UF MD mean**				0.918
Mean (SD)	0.719 (0.021)	0.719 (0.021)	0.719 (0.021)	
Range	0.676 - 0.824	0.669 - 0.775	0.669 - 0.824	
**Right UF RD mean**				0.953
Mean (SD)	0.532 (0.028)	0.532 (0.028)	0.532 (0.028)	
Range	0.474 - 0.641	0.477 - 0.621	0.474 - 0.641	
**Right UF Tractlength (mm)**				0.380
Mean (SD)	2442.187 (2332.3)	2185.145 (1371.9)	2341.941 (2012.8)	
Range	584.730 - 19304.0	641.030 - 10078.0	584.730 - 19304.0	
**Left Corticospinal FA mean**				0.011
# missing	1	0	1	
Mean (SD)	0.641 (0.023)	0.633 (0.024)	0.638 (0.024)	
Range	0.589 - 0.712	0.580 - 0.686	0.580 - 0.712	
**Left Corticospinal AD mean**				0.384
# missing	1	0	1	
Mean (SD)	1.072 (0.029)	1.076 (0.031)	1.074 (0.030)	

Range	0.972 - 1.157	1.009 - 1.169	0.972 - 1.169	
**Left Corticospinal MD mean**				< 0.001
# missing	1	0	1	
Mean (SD)	0.573 (0.014)	0.580 (0.012)	0.576 (0.014)	
Range	0.528 - 0.619	0.543 - 0.607	0.528 - 0.619	
**Left Corticospinal RD mean**				< 0.001
# missing	1	0	1	
Mean (SD)	0.323 (0.018)	0.332 (0.016)	0.327 (0.018)	
Range	0.275 - 0.380	0.297 - 0.367	0.275 - 0.380	
**Left Corticospinal Tractlength (mm)**				0.339
# missing	1	0	1	
Mean (SD)	2560.879 (1444.14)	2765.294 (1509.79)	2641.001 (1469.84)	
Range	1145.0 - 8438.70	1343.70 - 10183.0	1145.0 - 10183.0	
**Right Corticospinal FA mean**				0.087
# missing	1	0	1	
Mean (SD)	0.623 (0.026)	0.617 (0.025)	0.621 (0.026)	
Range	0.556 - 0.691	0.547 - 0.669	0.547 - 0.691	
**Right Corticospinal AD mean**				0.43
# missing	1	0	1	
Mean (SD)	1.082 (0.030)	1.086 (0.030)	1.083 (0.030)	
Range	1.001 - 1.170	1.011 - 1.175	1.001 - 1.175	
**Right Corticospinal MD mean**				0.003
# missing	1	0	1	
Mean (SD)	0.588 (0.014)	0.594 (0.011)	0.590 (0.013)	
Range	0.540 - 0.634	0.568 - 0.627	0.540 - 0.634	
**Right Corticospinal RD mean**				0.011
# missing	1	0	1	
Mean (SD)	0.341 (0.019)	0.348 (0.017)	0.344 (0.018)	
Range	0.294 - 0.387	0.315 - 0.396	0.294 - 0.396	
**Right Corticospinal Tractlength (mm)**				0.527

# missing	1	0	1	
Mean (SD)	1658.098 (961.90)	1748.389 (1008.2)	1693.488 (978.77)	
Range	421.060 - 8001.20	656.770 - 6862.50	1693.488 (978.77)	
**Left Cingulum Cingulate FA mean**				0.534
# missing	4	0	4	
Mean (SD)	0.509 (0.044)	0.509 (0.044)	0.507 (0.044)	
Range	0.361 - 0.609	0.352 - 0.602	0.352 - 0.609	
**Left Cingulum Cingulate AD mean**				0.695
# missing	4	0	4	
Mean (SD)	1.052 (0.040)	1.050 (0.045)	1.051 (0.042)	
Range	0.952 - 1.144	0.914 - 1.162	0.914 - 1.162	
**Left Cingulum Cingulate MD mean**				0.792
# missing	4	0	4	
Mean (SD)	0.641 (0.023)	0.642 (0.026)	0.642 (0.024)	
Range	0.590 - 0.708	0.593 - 0.711	0.590 - 0.711	
**Left Cingulum Cingulate RD mean**				0.603
# missing	4	0	4	
Mean (SD)	0.436 (0.034)	0.439 (0.035)	0.437 (0.034)	
Range	0.351 - 0.524	0.365 - 0.523	0.351 - 0.524	
**Left Cingulum Cingulate Tractlength (mm)**				0.177
# missing	2	0	2	
Mean (SD)	3594.148 (2077.607)	4082.2 (2995.012)	3786.420 (2484.08)	
Range	938.920 - 14609.0	1218.0 - 18612.0	938.920 - 18612.0	
**Right Cingulum Cingulate FA mean**				0.774
# missing	2	0	2	
Mean (SD)	0.469 (0.047)	0.467 (0.043)	0.469 (0.045)	
Range	0.336 - 0.613	0.340 - 0.580	0.336 - 0.613	
**Right Cingulum Cingulate AD mean**				0.674
# missing	2	0	2	

Mean (SD)	1.003 (0.041)	1.000 (0.039)	1.002 (0.040)	
Range	0.883 - 1.089	0.917 - 1.105	0.883 - 1.105	
**Right Cingulum Cingulate MD mean**				0.938
# missing	2	0	2	
Mean (SD)	0.636 (0.020)	0.636 (0.022)	0.636 (0.021)	
Range	0.595 - 0.694	0.585 - 0.698	0.585 - 0.698	
**Right Cingulum Cingulate RD mean**				0.853
# missing	2	0	2	
Mean (SD)	0.453 (0.033)	0.454 (0.032)	0.453 (0.032)	
Range	0.353 - 0.547	0.376 - 0.563	0.353 - 0.563	
**Right Cingulum Cingulate Tractlength (mm)**				0.145
Mean (SD)	3724.8 (3104.5)	3140.9 (2080.2)	3497.1 (2759.7)	
Range	554.820 - 29447.0	875.710 - 13884.0	554.820 - 29447.0	
**Left IFOF FA mean**				0.167
Mean (SD)	0.488 (0.027)	0.493 (0.031)	0.490 (0.029)	
Range	0.419 - 0.549	0.427 - 0.567	0.419 - 0.567	
**Left IFOF AD mean**				0.066
Mean (SD)	1.089 (0.034)	1.099 (0.041)	1.093 (0.037)	
Range	0.991 - 1.186	1.026 - 1.230	0.991 - 1.230	
**Left IFOF MD mean**				0.472
Mean (SD)	0.680 (0.020)	0.682 (0.023)	0.681 (0.021)	
Range	0.634 - 0.747	0.641 - 0.762	0.634 - 0.762	
**Left IFOF RD mean**				0.67
Mean (SD)	0.476 (0.024)	0.474 (0.027)	0.475 (0.025)	
Range	0.420 - 0.552	0.422 - 0.545	0.420 - 0.552	
**Left IFOF Tractlength (mm)**				0.346
Mean (SD)	6251.4 (3734.6)	6763.5 (3737.8)	6451.1 (3734.9)	
Range	1851.20 - 19454.0	2361.10 - 22823.0	1851.20 - 22823.0	
**Right IFOF FA mean**				0.316

Mean (SD)	0.491 (0.025)	0.495 (0.031)	0.492 (0.027)	
Range	0.423 - 0.548	0.415 - 0.557	0.415 - 0.557	
**Right IFOF AD mean**				0.068
Mean (SD)	1.089 (0.035)	1.099 (0.039)	1.093 (0.037)	
Range	1.020 - 1.172	0.989 - 1.223	0.989 - 1.223	
**Right IFOF MD mean**				0.327
Mean (SD)	0.679 (0.020)	0.682 (0.020)	0.680 (0.020)	
Range	0.637 - 0.756	0.640 - 0.730	0.637 - 0.756	
**Right IFOF RD mean**				0.865
Mean (SD)	0.474 (0.023)	0.473 (0.025)	0.474 (0.024)	
Range	0.414 - 0.561	0.423 - 0.531	0.414 - 0.561	
**Right IFOF Tractlength (mm)**				0.437
Mean (SD)	3761.091 (2190.41)	4044.9 (2948.31)	3871.8 (2510.0)	
Range	1599.6 - 15007.0	1938.70 - 18954.0	1599.60 - 18954.0	
**Motion During DTI Scan**				0.960
Mean (SD)	-0.052 (0.062)	-0.051 (0.053)	-0.052 (0.059)	
Range	-0.217 - 0.126	-0.158 - 0.092	-0.217 - 0.126	

Table S2A. Summary of estimated linear associations between neighborhood disadvantage and tract fractional anisotropy in left hemisphere. All linear models presented without covariates. All reported beta coefficients are standardized. CI=confidence interval; SE=standard error. AF=arcuate fasciculus; CC=cingulum cingulate; CST=corticospinal tract; FA=fractional anisotropy; FDR=false discovery rate; IFOF= inferior fronto-occiptal fasciculus; UF=uncinate fasciculus.

Tract	Beta Coefficient	SE	95% CI	*t*-value	*p*-value	FDR-corrected *p*-value	R^2	ΔR^2
L AF FA	-0.18	0.07	[-0.32, -0.05]	-2.62	0.009**	0.045*	0.034	0.034
L CC FA	0.02	0.07	[-0.12, 0.16]	0.23	0.817	0.817	0.00	0.0002
L CST FA	0.03	0.07	[-0.11, 0.17]	0.44	0.657	0.817	0.001	0.001
L IFOF FA	-0.07	0.07	[-0.21, 0.07]	-0.95	0.345	0.575	0.005	0.005
L UF FA	-0.15	0.07	[-0.29, -0.01]	-2.09	0.038*	0.095	0.022	0.022

Table S2B. Summary of estimated linear associations between neighborhood disadvantage and tract fractional anisotropy in right hemisphere. All linear models presented without covariates. All reported beta coefficients are standardized. CI=confidence interval; SE=standard error. AF=arcuate fasciculus; CC=cingulum cingulate; CST=corticospinal tract; FA=fractional anisotropy; FDR=false discovery rate; IFOF= inferior fronto-occiptal fasciculus; UF=uncinate fasciculus.

Tract	Beta Coefficient	SE	95% CI	*t*-value	*p*-value	FDR-corrected *p*-value	R^2	ΔR^2
R AF FA	-0.16	0.08	[-0.31, -0.01]	-2.12	0.036*	0.09	0.020	0.0253
R CC FA	-0.003	0.07	[-0.14, 0.14]	-0.04	0.971	0.971	0.00	0.00001
R CST FA	-0.03	0.07	[-0.17, 0.11]	-0.43	0.665	0.971	0.001	0.001
R IFOF FA	0.02	0.07	[-0.12, 0.16]	0.22	0.826	0.971	0.00	0.0002
R UF FA	-0.21	0.07	[-0.35, -0.08]	-3.05	0.003**	0.015*	0.045	0.045

Table S2C. Summary of estimated linear associations between neighborhood disadvantage and tract fractional anisotropy in left hemisphere using data from CalEnviroScreen4.0. In all linear models, age, sex, body mass index, depression severity, psychiatric medication use, study group, race, scan time point, tract length, motion during the scan, and parental education level were included as covariates. Neighborhood disadvantage is extracted from the CalEnviroScreen4.0. All reported beta coefficients are standardized. CI=confidence interval; SE=standard error. AF=arcuate fasciculus; CC=cingulum cingulate; FA=fractional anisotropy; FDR=false discovery rate; UF=uncinate fasciculus.

Tract	Beta Coefficient	SE	95% CI	*t*-value	*p*-value	FDR-corrected *p*-value	R^2	ΔR^2
L AF FA	-0.18	0.09	[-0.35, -0.01]	-2.07	0.04*	0.123	0.148	0.0256
L CC FA	-0.14	0.08	[-0.30, 0.03]	-1.67	0.096	0.16	0.213	0.017
L CST FA	-0.08	0.08	[-0.24, 0.09]	-0.91	0.366	0.366	0.193	0.005
L IFOF FA	-0.08	0.09	[-0.26, 0.09]	-0.97	0.333	0.366	0.135	0.0057
L UF FA	-0.17	0.09	[-0.34, 0.00]	-1.98	0.049*	0.123	0.149	0.0235

Table S2D. Summary of estimated linear associations between neighborhood disadvantage and tract fractional anisotropy in right hemisphere using data from the CalEnviroScreen4.0. In all linear models, age, sex, body mass index, depression severity, psychiatric medication use, study group, race, scan time point, tract length, motion during the scan, and parental education level were included as covariates. Neighborhood disadvantage is extracted from the CalEnviroScreen4.0. All reported beta coefficients are standardized. CI=confidence interval; SE=standard error. AF=arcuate fasciculus; CC=cingulum cingulate; FA=fractional anisotropy; FDR=false discovery rate; UF=uncinate fasciculus.

Tract	Beta Coefficient	SE	95% CI	*t*-value	*p*-value	FDR-corrected *p*-value	R^2	ΔR^2
R AF FA	-0.19	0.1	[-0.38, 0.01]	-1.92	0.056	0.14	0.100	0.025
R CC FA	-0.02	0.09	[-0.20, 0.15]	-0.28	0.779	0.973	0.133	0.0005
R CST FA	-0.13	0.08	[-0.29, 0.03]	-1.59	0.113	0.188	0.255	0.015
R IFOF FA	-0.003	0.08	[-0.17, 0.16]	-0.03	0.973	0.973	0.194	0.00007
R UF FA	-0.27	0.09	[-0.44, -0.10]	-3.08	0.002**	0.01*	0.125	0.0542

Table S3A. Summary of estimated linear associations between parental education level and tract fractional anisotropy in left hemisphere. In all linear models, age, sex, body mass index, psychiatric medication use, depression severity, study group, race, scan time point, tract length, and motion during the scan, were included as covariates. All reported beta coefficients are standardized. CI=confidence interval; SE=standard error. AF=arcuate fasciculus; CC=cingulum cingulate; CST=corticospinal tract; FA=fractional anisotropy; FDR=false discovery rate; IFOF= inferior fronto- occipital fasciculus; UF=uncinate fasciculus.

**Tract**	**Beta Coefficient**	**SE**	**95% CI**	***t*-value**	***p*-value**	**FDR-corrected *p*-value**	R^2	ΔR^2
L AF FA	-0.03	0.76	[-1.53, 1.48]	-0.04	0.972	0.972	0.122	0.011
L CC FA	0.34	0.60	[-0.85, 1.53]	0.56	0.575	0.719	0.199	0.022
L CST FA	-0.55	0.60	[-1.74, 0.64]	-0.91	0.365	0.608	0.187	0.037
L IFOF FA	-0.76	0.62	[-1.99, 048]	-1.21	0.228	0.57	0.130	0.025
L UF FA	-1.29	0.63	[-2.52, -0.05]	-2.06	0.041*	0.205	0.130	0.042

Table S3B. Summary of estimated linear associations between parental education level and tract fractional anisotropy in left hemisphere. All linear models reported without covariates. All reported beta coefficients are standardized. CI=confidence interval; SE=standard error. AF=arcuate fasciculus; CC=cingulum cingulate; CST=corticospinal tract; FA=fractional anisotropy; FDR=false discovery rate; IFOF= inferior fronto-occiptal fasciculus; UF=uncinate fasciculus.

**Tract**	**Beta Coefficient**	**SE**	**95% CI**	***t*-value**	***p*-value**	**FDR-corrected *p*-value**	R^2	ΔR^2
L AF FA	0.06	0.75	[-1.41, 1.53]	0.08	0.935	0.935	0.008	0.008
L CC FA	0.33	0.61	[-0.88, 1.54]	0.54	0.590	0.738	0.036	0.036
L CST FA	-0.53	0.62	[-1.75, 0.70]	-0.85	0.398	0.663	0.022	0.022
L IFOF FA	-0.63	0.62	[-1.85, 0.60]	-1.01	0.314	0.663	0.011	0.011
L UF FA	-1.18	0.62	[-2.39, 0.03]	-1.92	0.056	0.28	0.033	0.033

Table S3C. Summary of estimated linear associations between parental education level and tract fractional anisotropy in right hemisphere. In all linear models, age, sex, body mass index, psychiatric medication use, depression severity, study group, race, scan time point, tract length, and motion during the scan, were included as covariates. All reported beta coefficients are standardized. CI=confidence interval; SE=standard error. AF=arcuate fasciculus; CC=cingulum cingulate; CST=corticospinal tract; FA=fractional anisotropy; FDR=false discovery rate; IFOF= inferior fronto-occipital fasciculus; UF=uncinate fasciculus.

Tract	Beta Coefficient	SE	95% CI	*t*-value	*p*-value	FDR-corrected *p*-value	R^2	ΔR^2
R AF FA	-1.39	1.09	[-3.54, 0.77]	-1.27	0.205	0.513	0.071	0.014
R CC FA	-0.29	0.63	[-1.53, 0.95]	-0.46	0.647	0.801	0.123	0.023
R CST FA	-0.15	0.59	[-1.31, 1.01]	-0.25	0.801	0.801	0.243	0.015
R IFOF FA	-0.62	0.61	[-1.83, 0.58]	-1.02	0.311	0.518	0.191	0.031
R UF FA	-0.89	0.64	[-2.16, 0.38]	-1.38	0.171	0.513	0.079	0.016

Table S3D. Summary of estimated linear associations between parental education level and tract fractional anisotropy in right hemisphere. All linear models reported without covariates. All reported beta coefficients are standardized. CI=confidence interval; SE=standard error. AF=arcuate fasciculus; CC=cingulum cingulate; CST=corticospinal tract; FA=fractional anisotropy; FDR=false discovery rate; IFOF= inferior fronto-occiptal fasciculus; UF=uncinate fasciculus.

Tract	Beta Coefficient	SE	95% CI	*t*-value	*p*-value	FDR-corrected *p*-value	R^2	ΔR^2
R AF FA	-1.59	1.03	[-3.62, 0.45]	-1.54	0.126	0.338	0.018	0.018
R CC FA	-0.31	0.62	[-1.54, 0.91]	-0.50	0.616	0.77	0.012	0.012
R CST FA	-0.18	0.61	[-1.39, 1.03]	-0.29	0.771	0.77	0.04	0.04
R IFOF FA	-0.36	0.62	[-1.59, 0.86]	-0.58	0.560	0.77	0.016	0.016
R UF FA	-0.93	0.62	[-2.15, 0.29]	-1.50	0.135	0.338	0.018	0.018

Table S4A. Summary of estimated linear associations between higher education disadvantage percentiles and tract fractional anisotropy in left hemisphere. In all linear models, age, sex, body mass index, psychiatric medication use, depression severity, study group, race, scan time point, tract length, and motion during the scan, were included as covariates. All reported beta coefficients are standardized. CI=confidence interval; SE=standard error. AF=arcuate fasciculus; CC=cingulum cingulate; CST=corticospinal tract; FA=fractional anisotropy; FDR=false discovery rate; IFOF= inferior fronto-occipital fasciculus; UF=uncinate fasciculus.

Tract	Beta Coefficient	SE	95% CI	*t*-value	*p*-value	FDR-corrected *p*-value	R^2	ΔR^2
L AF FA	-0.22	0.08	[-0.38, -0.06]	-2.70	0.008**	0.028*	0.167	0.043
L CC FA	-0.21	0.08	[-0.37, -0.05]	-2.57	0.011*	0.028*	0.231	0.040
L CST FA	-0.11	0.08	[-0.28, 0.05]	-1.39	0.167	0.167	0.201	0.012
L IFOF FA	-0.19	0.08	[-0.36, -0.03]	-2.28	0.024*	0.04*	0.151	0.031
L UF FA	-0.16	0.09	[-0.32, 0.01]	-1.82	0.07	0.088	0.144	0.020

Table S4B. Summary of estimated linear associations between higher education disadvantage percentiles and tract fractional anisotropy in left hemisphere. All linear models reported without any covariates. All reported beta coefficients are standardized. CI=confidence interval; SE=standard error. AF=arcuate fasciculus; CC=cingulum cingulate; CST=corticospinal tract; FA=fractional anisotropy; FDR=false discovery rate; IFOF= inferior fronto-occiptal fasciculus; UF=uncinate fasciculus.

Tract	Beta Coefficient	SE	95% CI	*t*-value	*p*-value	FDR-corrected *p*-value	R^2	ΔR^2
L AF FA	-0.19	0.07	[-0.33, -0.05]	-2.67	0.008**	0.04*	0.036	0.036
L CC FA	-0.007	0.07	[-0.15, 0.14]	-0.10	0.922	0.922	0.00	0.000051
L CST FA	-0.02	0.07	[-0.16, 0.12]	-0.26	0.799	0.922	0.00	0.000034
L IFOF FA	-0.08	0.07	[-0.22, 0.06]	-1.11	0.268	0.447	0.006	0.006
L UF FA	-0.11	0.07	[-0.25, 0.03]	-1.52	0.131	0.328	0.012	0.012

Table S4C. Summary of estimated linear associations between higher education disadvantage percentiles and tract fractional anisotropy in left hemisphere in CalEnviroScreen4.0 data. In all linear models, age, sex, body mass index, depression severity, psychiatric medication use, study group, race, scan time point, tract length, motion during the scan, and parental education level were included as covariates. Higher education disadvantage is extracted from the CalEnviroScreen4.0. All reported beta coefficients are standardized. CI=confidence interval; SE=standard error. AF=arcuate fasciculus; CC=cingulum cingulate; FA=fractional anisotropy; FDR=false discovery rate; UF=uncinate fasciculus.

Tract	Beta Coefficient	SE	95% CI	*t*-value	*p*-value	FDR-corrected *p*-value	R^2	ΔR^2
L AF FA	-0.21	0.08	[-0.37, -0.05]	-2.55	0.012*	0.03*	0.158	0.0381
L CC FA	-0.22	0.08	[-0.38, 0.06]	-2.73	0.007**	0.03*	0.140	0.044
L CST FA	-0.12	0.08	[-0.27, 0.04]	-1.43	0.154	0.193	0.199	0.0124
L IFOF FA	-0.15	0.08	[-0.31, 0.01]	-1.80	0.074	0.123	0.146	0.0192
L UF FA	-0.10	0.08	[-0.26, 0.07]	-1.17	0.244	0.244	0.136	0.0083

Table S4D. Summary of estimated linear associations between higher education disadvantage percentile and tract fractional anisotropy in right hemisphere. In all linear models, age, sex, body mass index, psychiatric medication use, depression severity, study group, race, scan time point, tract length, and motion during the scan, were included as covariates. All reported beta coefficients are standardized. CI=confidence interval; SE=standard error. AF=arcuate fasciculus; CC=cingulum cingulate; CST=corticospinal tract; FA=fractional anisotropy; FDR=false discovery rate; IFOF= inferior fronto-occipital fasciculus; UF=uncinate fasciculus.

Tract	Beta Coefficient	SE	95% CI	*t*-value	*p*-value	FDR-corrected *p*-value	R^2	ΔR^2
R AF FA	-0.22	0.09	[-0.41, -0.04]	-2.37	0.019*	0.095	0.102	0.040
R CC FA	-0.11	0.09	[-0.28, 0.06]	-1.24	0.217	0.271	0.146	0.010
R CST FA	-0.14	0.08	[-0.30, 0.01]	-1.79	0.075	0.125	0.261	0.019
R IFOF FA	-0.09	0.08	[-0.25, 0.08]	-1.04	0.299	0.299	0.196	0.007
R UF FA	-0.17	0.09	[-0.34, 0.00]	-1.94	0.054	0.125	0.094	0.022

Table S4E. Summary of estimated linear associations between higher education disadvantage percentile and tract fractional anisotropy in right hemisphere. All linear models reported without any covariates. All reported beta coefficients are standardized. CI=confidence interval; SE=standard error. AF=arcuate fasciculus; CC=cingulum cingulate; CST=corticospinal tract; FA=fractional anisotropy; FDR=false discovery rate; IFOF= inferior fronto-occipital fasciculus; UF=uncinate fasciculus.

Tract	Beta Coefficient	SE	95% CI	*t*-value	*p*-value	FDR-corrected *p*-value	R^2	ΔR^2
R AF FA	-0.19	0.08	[-0.34, -0.04]	-2.52	0.013*	0.065	0.036	0.036
R CC FA	-0.03	0.07	[-0.17, 0.11]	-0.40	0.693	0.866	0.001	0.001
R CST FA	-0.06	0.07	[-0.20, 0.08]	-0.88	0.378	0.63	0.004	0.004
R IFOF FA	0.01	0.07	[-0.13, 0.15]	0.14	0.889	0.889	0.00	0.0001
R UF FA	-0.11	0.07	[-0.25, 0.03]	-1.56	0.121	0.303	0.012	0.012

Table S4F. Summary of estimated linear associations between higher education disadvantage percentiles and tract fractional anisotropy in right hemisphere in CalEnviroScreen4.0 data. In all linear models, age, sex, body mass index, depression severity, psychiatric medication use, study group, race, scan time point, tract length, motion during the scan, and parental education level were included as covariates. Higher education disadvantage is extracted from the CalEnviroScreen4.0. All reported beta coefficients are standardized. CI=confidence interval; SE=standard error. AF=arcuate fasciculus; CC=cingulum cingulate; FA=fractional anisotropy; FDR=false discovery rate; UF=uncinate fasciculus.

Tract	Beta Coefficient	SE	95% CI	*t*-value	*p*-value	FDR-corrected *p*-value	R^2	ΔR^2
R AF FA	-0.13	0.09	[-0.30, -0.04]	-1.57	0.119	0.198	0.111	0.038
R CC FA	-0.10	0.08	[-0.27, 0.07]	-1.18	0.238	0.298	0.140	0.0085
R CST FA	-0.13	0.08	[-0.28, 0.03]	-1.63	0.104	0.198	0.265	0.016
R IFOF FA	-0.04	0.08	[-0.20, 0.12]	-0.47	0.642	0.642	0.195	0.00132
R UF FA	-0.22	0.09	[-0.39, 0.04]	-2.37	0.019*	0.095	0.088	0.015

Table S5A. Summary of estimated linear associations between higher poverty disadvantage percentiles and tract fractional anisotropy in left hemisphere. In all linear models, age, sex, body mass index, psychiatric medication use, depression severity, study group, race, scan time point, tract length, and motion during the scan, were included as covariates. All reported beta coefficients are standardized. CI=confidence interval; SE=standard error. AF=arcuate fasciculus; CC=cingulum cingulate; CST=corticospinal tract; FA=fractional anisotropy; FDR=false discovery rate; IFOF= inferior fronto-occipital fasciculus; UF=uncinate fasciculus.

Tract	Beta Coefficient	SE	95% CI	*t*-value	*p*-value	FDR-corrected *p*-value	R^2	ΔR^2
L AF FA	-0.21	0.08	[-0.37, -0.06]	-2.68	0.008**	0.02*	0.159	0.042
L CC FA	-0.15	0.08	[-0.30, 0.01]	-1.91	0.058	0.073	0.217	0.022
L CST FA	-0.09	0.08	[-0.25, 0.06]	-1.22	0.225	0.225	0.194	0.001
L IFOF FA	-0.17	0.08	[-0.32, -0.01]	-2.08	0.039*	0.065	0.152	0.025
L UF FA	-0.24	0.08	[-0.39, -0.08]	-2.99	0.003**	0.015*	0.175	0.052

Table S5B. Summary of estimated linear associations between higher poverty disadvantage percentiles and tract fractional anisotropy in left hemisphere. All linear models reported without any covariates. All reported beta coefficients are standardized. CI=confidence interval; SE=standard error. AF=arcuate fasciculus; CC=cingulum cingulate; CST=corticospinal tract; FA=fractional anisotropy; FDR=false discovery rate; IFOF= inferior fronto-occiptal fasciculus; UF=uncinate fasciculus.

Tract	Beta Coefficient	SE	95% CI	*t*-value	*p*-value	FDR-corrected *p*-value	R^2	ΔR^2
L AF FA	-0.18	0.07	[-0.31, -0.04]	-2.50	0.013*	0.033*	0.031	0.031
L CC FA	-0.3	0.07	[-0.18, 0.11]	-0.47	0.640	0.8	0.001	0.001
L CST FA	-0.01	0.07	[-0.15, 0.13]	-0.17	0.866	0.866	0.00	0.0002
L IFOF FA	-0.08	0.07	[-0.22, 0.06]	-1.09	0.277	0.462	0.006	0.006
L UF FA	-0.19	0.07	[-0.33, -0.05]	-2.67	0.008**	0.033*	0.035	0.035

Table S5C. Summary of estimated linear associations between poverty disadvantage percentiles and tract fractional anisotropy in left hemisphere from CalEnviroScreen4.0 data. In all linear models, age, sex, body mass index, depression severity, psychiatric medication use, study group, race, scan time point, tract length, motion during the scan, and parental education level were included as covariates. Higher poverty disadvantage is extracted from the CalEnviroScreen4.0. All reported beta coefficients are standardized. CI=confidence interval; SE=standard error. AF=arcuate fasciculus; CC=cingulum cingulate; FA=fractional anisotropy; FDR=false discovery rate; UF=uncinate fasciculus.

Tract	Beta Coefficient	SE	95% CI	*t*-value	*p*-value	FDR-corrected *p*-value	R^2	ΔR^2
L AF FA	-0.23	0.08	[-0.39, 0.08]	-2.95	0.004**	0.013*	0.166	0.0503
L CC FA	-0.14	0.08	[-0.29, 0.02]	-1.76	0.08	0.1	0.214	0.0187
L CST FA	-0.05	0.08	[-0.21, 0.10]	-0.67	0.506	0.506	0.189	0.0027
L IFOF FA	-0.22	0.08	[-0.38, -0.07]	-2.82	0.005**	0.13*	0.170	0.046
L UF FA	-0.20	0.08	[-0.35, 0.04]	-2.50	0.014*	0.023*	0.161	0.0366

Table S5D. Summary of estimated linear associations between higher poverty disadvantage percentile and tract fractional anisotropy in right hemisphere. In all linear models, age, sex, body mass index, psychiatric medication use, depression severity, study group, race, scan time point, tract length, and motion during the scan, were included as covariates. All reported beta coefficients are standardized. CI=confidence interval; SE=standard error. AF=arcuate fasciculus; CC=cingulum cingulate; CST=corticospinal tract; FA=fractional anisotropy; FDR=false discovery rate; IFOF= inferior fronto-occipital fasciculus; UF=uncinate fasciculus.

Tract	Beta Coefficient	SE	95% CI	*t*-value	*p*-value	FDR-corrected *p*-value	R^2	ΔR^2
R AF FA	-0.17	0.09	[-0.35, 0.00]	-1.94	0.054	0.135	0.095	0.025
R CC FA	-0.06	0.08	[-0.22, 0.10]	-0.77	0.443	0.554	0.126	0.004
R CST FA	-0.11	0.08	[-0.26, 0.04]	-1.46	0.147	0.245	0.253	0.013
R IFOF FA	-0.03	0.08	[-0.19, 0.12]	-0.45	0.657	0.657	0.192	0.001
R UF FA	-0.26	0.08	[-0.42, -0.10]	-3.19	0.002**	0.01*	0.133	0.058

Table S5E. Summary of estimated linear associations between higher poverty disadvantage percentile and tract fractional anisotropy in right hemisphere. All linear models reported without any covariates. All reported beta coefficients are standardized. CI=confidence interval; SE=standard error. AF=arcuate fasciculus; CC=cingulum cingulate; CST=corticospinal tract; FA=fractional anisotropy; FDR=false discovery rate; IFOF= inferior fronto-occiptal fasciculus; UF=uncinate fasciculus.

Tract	Beta Coefficient	SE	95% CI	*t*-value	*p*-value	FDR-corrected *p*-value	R^2	ΔR^2
R AF FA	-0.15	0.07	[-0.30, 0.00]	-1.97	0.051	0.128	0.022	0.022
R CC FA	-0.03	0.07	[-0.17, 0.11]	-0.38	0.708	0.708	0.001	0.001
R CST FA	-0.04	0.07	[-0.18, 0.10]	-0.61	0.543	0.708	0.002	0.002
R IFOF FA	0.04	0.07	[-0.10, 0.18]	0.54	0.589	0.708	0.001	0.0015
R UF FA	-0.20	0.07	[-0.33, -0.06]	-2.82	0.005**	0.03*	0.039	0.039

Table S5F. Summary of estimated linear associations between poverty disadvantage percentiles and tract fractional anisotropy in right hemisphere from CalEnviroScreen4.0 data. In all linear models, age, sex, body mass index, depression severity, psychiatric medication use, study group, race, scan time point, tract length, motion during the scan, and parental education level were included as covariates. Higher poverty disadvantage is extracted from the CalEnviroScreen4.0. All reported beta coefficients are standardized. CI=confidence interval; SE=standard error. AF=arcuate fasciculus; CC=cingulum cingulate; FA=fractional anisotropy; FDR=false discovery rate; UF=uncinate fasciculus.

Tract	Beta Coefficient	SE	95% CI	*t*-value	*p*-value	FDR-corrected *p*-value	R^2	ΔR^2
R AF FA	-0.14	0.09	[-0.32, 0.03]	-1.62	0.107	0.268	0.088	0.0179
R CC FA	-0.06	0.08	[-0.22, 0.10]	-0.79	0.431	0.431	0.126	0.0038
R CST FA	-0.09	0.08	[-0.24, 0.06]	-1.19	0.234	0.355	0.250	0.0086
R IFOF FA	-0.08	0.08	[-0.24, 0.07]	-1.08	0.284	0.355	0.197	0.0069
R UF FA	-0.22	0.08	[-0.38, 0.06]	-2.74	0.007**	0.035*	0.119	0.0432

Table S6A. Summary of estimated linear associations between higher unemployment disadvantage percentiles and tract fractional anisotropy in left hemisphere. In all linear models, age, sex, body mass index, psychiatric medication use, depression severity, study group, race, scan time point, tract length, and motion during the scan, were included as covariates. All reported beta coefficients are standardized. CI=confidence interval; SE=standard error. AF=arcuate fasciculus; CC=cingulum cingulate; CST=corticospinal tract; FA=fractional anisotropy; FDR=false discovery rate; IFOF= inferior fronto-occipital fasciculus; UF=uncinate fasciculus.

Tract	Beta Coefficient	SE	95% CI	*t*-value	*p*-value	FDR-corrected *p*-value	R^2	ΔR^2
L AF FA	-0.15	0.08	[-0.31, 0.00]	-1.95	0.053	0.265	0.142	0.022
L CC FA	0.02	0.08	[-0.14, 0.17]	0.20	0.839	0.839	0.199	0.0001
L CST FA	0.02	0.76	[-0.13, 0.17]	0.28	0.779	0.839	0.187	0.0004
L IFOF FA	-0.08	0.08	[-0.24, 0.07]	-1.05	0.295	0.738	0.136	0.007
L UF FA	-0.02	0.08	[-0.18, 0.13]	-0.29	0.775	0.839	0.130	0.005

Table S6B. Summary of estimated linear associations between higher unemployment disadvantage percentiles and tract fractional anisotropy in left hemisphere. All linear models reported without any covariates. All reported beta coefficients are standardized. CI=confidence interval; SE=standard error. AF=arcuate fasciculus; CC=cingulum cingulate; CST=corticospinal tract; FA=fractional anisotropy; FDR=false discovery rate; IFOF= inferior fronto-occiptal fasciculus; UF=uncinate fasciculus.

Tract	Beta Coefficient	SE	95% CI	*t*-value	*p*-value	FDR-corrected *p*-value	R^2	ΔR^2
L AF FA	-0.15	0.07	[-0.29, -0.01]	-2.11	0.036*	0.18	0.022	0.022
L CC FA	0.08	0.07	[-0.6, 0.23]	1.17	0.242	0.403	0.007	0.007
L CST FA	0.12	0.07	[-0.02, 0.26]	1.64	0.103	0.258	0.013	0.013
L IFOF FA	-0.04	0.07	[-0.18, 0.10]	-0.57	0.568	0.71	0.002	0.002
L UF FA	-0.02	0.07	[-0.16, 0.12]	-0.31	0.756	0.756	0.00	0.0005

Table S6C. Summary of estimated linear associations between higher unemployment disadvantage percentile and tract fractional anisotropy in right hemisphere. In all linear models, age, sex, body mass index, psychiatric medication use, depression severity, study group, race, scan time point, tract length, and motion during the scan, were included as covariates. All reported beta coefficients are standardized. CI=confidence interval; SE=standard error. AF=arcuate fasciculus; CC=cingulum cingulate; CST=corticospinal tract; FA=fractional anisotropy; FDR=false discovery rate; IFOF= inferior fronto-occipital fasciculus; UF=uncinate fasciculus.

Tract	Beta Coefficient	SE	95% CI	*t*-value	*p*-value	FDR-corrected *p*-value	R^2	ΔR^2
R AF FA	-0.03	0.09	[-0.20, 0.14]	-0.32	0.752	0.752	0.072	0.0007
R CC FA	0.07	0.08	[-0.09, 0.23]	0.85	0.395	0.747	0.127	0.004
R CST FA	-0.06	0.07	[-0.20. 0.09]	-0.76	0.448	0.747	0.246	0.003
R IFOF FA	-0.04	0.08	[-0.19, 0.11]	-0.51	0.610	0.752	0.192	0.002
R UF FA	-0.12	0.08	[-0.28, 0.04]	-1.49	0.138	0.690	0.091	0.013

Table S6D. Summary of estimated linear associations between higher unemployment disadvantage percentile and tract fractional anisotropy in right hemisphere. All linear models reported without any covariates. All reported beta coefficients are standardized. CI=confidence interval; SE=standard error. AF=arcuate fasciculus; CC=cingulum cingulate; CST=corticospinal tract; FA=fractional anisotropy; FDR=false discovery rate; IFOF= inferior fronto-occiptal fasciculus; UF=uncinate fasciculus.

Tract	Beta Coefficient	SE	95% CI	*t*-value	*p*-value	FDR-corrected *p*-value	R^2	ΔR^2
R AF FA	-0.04	0.08	[-0.19, 0.11]	-0.56	0.576	0.746	0.002	0.002
R CC FA	0.10	0.07	[-0.04, 0.24]	1.44	0.152	0.57	0.010	0.010
R CST FA	0.03	0.07	[-0.11, 0.17]	0.45	0.652	0.746	0.001	0.001
R IFOF FA	0.02	0.07	[-0.12, 0.16}	0.32	0.746	0.746	0.001	0.001
R UF FA	-0.09	0.07	[-0.23, 0.05]	-1.21	0.228	0.57	0.007	0.007

Table S7A. Summary of estimated linear associations between higher housing burden percentiles and tract fractional anisotropy (FA) in left hemisphere white matter tracts. In all linear models, age, sex, body mass index, psychiatric medication use, depression severity, study group, race, scan time point, tract length, and motion during the scan, were included as covariates. All reported beta coefficients are standardized. CI=confidence interval; SE=standard error. AF=arcuate fasciculus; CC=cingulum cingulate; CST=corticospinal tract; FA=fractional anisotropy; FDR=false discovery rate; IFOF= inferior fronto-occipital fasciculus; UF=uncinate fasciculus.

Tract	Beta Coefficient	SE	95% CI	*t*-value	*p*-value	FDR-corrected *p*-value	R^2	ΔR^2
L AF FA	-0.08	0.08	[-0.24, 0.08]	-1.02	0.309	0.515	0.131	0.006
L CC FA	-0.11	0.08	[-0.27, 0.05]	-1.38	0.170	0.425	0.208	0.011
L CST FA	0.05	0.08	[-0.11. 0.21]	0.65	0.519	0.525	0.191	0.002
L IFOF FA	-0.05	0.08	[-0.22, 0.11]	-0.64	0.525	0.525	0.132	0.003
L UF FA	-0.12	0.08	[-0.28, 0.05]	-1.41	0.160	0.425	0.139	0.012

Table S7B. Summary of estimated linear associations between higher housing burden percentiles and tract fractional anisotropy (FA) in left hemisphere white matter tracts. All linear models reported without any covariates. All reported beta coefficients are standardized. CI=confidence interval; SE=standard error. AF=arcuate fasciculus; CC=cingulum cingulate; CST=corticospinal tract; FA=fractional anisotropy; FDR=false discovery rate; IFOF= inferior fronto-occiptal fasciculus; UF=uncinate fasciculus.

Tract	Beta Coefficient	SE	95% CI	*t*-value	*p*-value	FDR-corrected *p*-value	R^2	ΔR^2
L AF FA	-0.12	0.07	[-0.26, 0.02]	-1.75	0.082	0.312	0.015	0.015
L CC FA	-0.04	0.07	[-0.18, 0.10]	-0.60	0.550	0.688	0.002	0.002
L CST FA	0.10	0.07	[-0.04, 0.24]	1.43	0.155	0.312	0.01	0.01
L IFOF FA	0.003	0.07	[-0.14, 0.14]	-0.04	0.971	0.971	0.00	0.00001
L UF FA	-0.09	0.07	[-0.24, 0.05]	-1.32	0.187	0.312	0.009	0.009

Table S7C. Summary of estimated linear associations between higher housing burden percentile and tract fractional anisotropy (FA) in right hemisphere white matter tracts. In all linear models, age, sex, body mass index, psychiatric medication use, depression severity, study group, race, scan time point, tract length, and motion during the scan, were included as covariates. All reported beta coefficients are standardized. CI=confidence interval; SE=standard error. AF=arcuate fasciculus; CC=cingulum cingulate; CST=corticospinal tract; FA=fractional anisotropy; FDR=false discovery rate; IFOF= inferior fronto- occipital fasciculus; UF=uncinate fasciculus.

Tract	Beta Coefficient	SE	95% CI	*t*-value	*p*-value	FDR-corrected *p*-value	R^2	ΔR^2
R AF FA	-0.11	0.09	[-0.28, 0.07]	-1.21	0.228	0.570	0.085	0.010
R CC FA	0.02	0.08	[-0.14, 0.18]	0.23	0.819	0.819	0.133	0.0003
R CST FA	-0.02	0.08	[-0.17, 0.14]	-0.24	0.810	0.803	0.244	0.0004
R IFOF FA	0.06	0.08	[-0.10, 0.21]	0.71	0.482	0.803	0.197	0.003
R UF FA	-0.16	0.08	[-0.33, 0.00]	-1.92	0.057	0.285	0.095	0.022

Table S7D. Summary of estimated linear associations between higher housing burden percentile and tract fractional anisotropy in right hemisphere. All linear models reported without any covariates. All reported beta coefficients are standardized. CI=confidence interval; SE=standard error. AF=arcuate fasciculus; CC=cingulum cingulate; CST=corticospinal tract; FA=fractional anisotropy; FDR=false discovery rate; IFOF= inferior fronto-occiptal fasciculus; UF=uncinate fasciculus.

Tract	Beta Coefficient	SE	95% CI	*t*-value	*p*-value	FDR-corrected *p*-value	R^2	ΔR^2
R AF FA	-0.08	0.08	[-0.23, 0.07]	-1.09	0.278	0.650	0.007	0.007
R CC FA	0.03	0.07	[-0.11, 0.17]	0.45	0.657	0.786	0.001	0.001
R CST FA	-0.02	0.07	[-0.16, 0.12]	-0.27	0.786	0.786	0.00	0.0004
R IFOF FA	0.06	0.07	[-0.08, 0.20]	0.86	0.390	0.650	0.004	0.004
R UF FA	-0.11	0.07	[-0.25, 0.03]	-1.60	0.111	0.555	0.013	0.013

Table S8A. Summary of estimated linear associations between higher linguistic isolation percentiles and tract fractional anisotropy in left hemisphere. In all linear models, age, sex, body mass index, psychiatric medication use, depression severity, study group, race, scan time point, tract length, and motion during the scan, were included as covariates. All reported beta coefficients are standardized. CI=confidence interval; SE=standard error. AF=arcuate fasciculus; CC=cingulum cingulate; CST=corticospinal tract; FA=fractional anisotropy; FDR=false discovery rate; IFOF= inferior fronto-occipital fasciculus; UF=uncinate fasciculus.

Tract	Beta Coefficient	SE	95% CI	*t*-value	*p*-value	FDR-corrected *p*-value	R^2	ΔR^2
L AF FA	-0.16	0.08	[-0.32. 0.00]	-2.03	0.044*	0.11	0.149	0.025
L CC FA	-0.20	0.08	[-0.35, -0.05]	-2.61	0.010*	0.05	0.249	0.040
L CST FA	-0.08	0.08	[-0.23, 0.08]	-1.00	0.317	0.414	0.192	0.006
L IFOF FA	-0.08	0.08	[-0.24, 0.08]	-0.98	0.331	0.414	0.131	0.006
L UF FA	-0.0005	0.08	[-0.16, 0.16]	-0.006	0.995	0.995	0.122	0.000

Table S8B. Summary of estimated linear associations between higher linguistic isolation percentiles and tract fractional anisotropy in left hemisphere. All linear models reported without any covariates. All reported beta coefficients are standardized. CI=confidence interval; SE=standard error. AF=arcuate fasciculus; CC=cingulum cingulate; CST=corticospinal tract; FA=fractional anisotropy; FDR=false discovery rate; IFOF= inferior fronto-occiptal fasciculus; UF=uncinate fasciculus.

Tract	Beta Coefficient	SE	95% CI	*t*-value	*p*-value	FDR-corrected *p*-value	R^2	ΔR^2
L AF FA	-0.20	0.07	[-0.34, -0.06]	-2.82	0.005**	0.025*	0.039	0.039
L CC FA	-0.09	0.07	[-0.23, 0.05]	-1.29	0.198	0.492	0.009	0.009
L CST FA	-0.04	0.07	[-0.18, 0.10]	-0.54	0.593	0.633	0.001	0.001
L IFOF FA	-0.07	0.07	[-0.22, 0.07]	-1.05	0.295	0.492	0.006	0.006
L UF FA	-0.03	0.07	[-0.18, 0.11]	-0.48	0.633	0.633	0.001	0.001

Table S8C. Summary of estimated linear associations between higher linguistic isolation percentile and tract fractional anisotropy in right hemisphere. In all linear models, age, sex, body mass index, psychiatric medication use, depression severity, study group, race, scan time point, tract length, and motion during the scan, were included as covariates. All reported beta coefficients are standardized. CI=confidence interval; SE=standard error. AF=arcuate fasciculus; CC=cingulum cingulate; CST=corticospinal tract; FA=fractional anisotropy; FDR=false discovery rate; IFOF= inferior fronto-occipital fasciculus; UF=uncinate fasciculus.

Tract	Beta Coefficient	SE	95% CI	*t*-value	*p*-value	FDR-corrected *p*-value	R^2	ΔR^2
R AF FA	-0.15	0.09	[-0.33, 0.03]	-1.60	0.112	0.476	0.079	0.018
R CC FA	-0.09	0.08	[-0.25, 0.07]	-1.10	0.275	0.458	0.154	0.007
R CST FA	-0.10	0..08	[-0.25, 0.05]	-1.34	0.183	0.476	0.251	0.011
R IFOF FA	0.04	0.08	[-0.12, 0.19]	0.47	0.642	0.775	0.196	0.001
R UF FA	-0.02	0.08	[-0.19, 0.14]	-0.29	0.775	0.775	0.06	0.0005

Table S8D. Summary of estimated linear associations between higher linguistic isolation percentile and tract fractional anisotropy in right hemisphere. All linear models reported without any covariates. All reported beta coefficients are standardized. CI=confidence interval; SE=standard error. AF=arcuate fasciculus; CC=cingulum cingulate; CST=corticospinal tract; FA=fractional anisotropy; FDR=false discovery rate; IFOF= inferior fronto-occiptal fasciculus; UF=uncinate fasciculus.

Tract	Beta Coefficient	SE	95% CI	*t*-value	*p*-value	FDR-corrected *p*-value	R^2	ΔR^2
R AF FA	-0.13	0.08	[-0.28, 0.02]	-1.67	0.097	0.485	0.016	0.016
R CC FA	-0.04	0.07	[-0.18, 0.10]	-0.60	0.551	0.899	0.002	0.002
R CST FA	-0.08	0.07	[-0.22, 0.06]	-1.14	0.255	0.638	0.007	0.007
R IFOF FA	0.007	0.07	[-0.13, 0.15]	0.10	0.924	0.924	0.00	0.00005
R UF FA	-0.03	0.07	[-0.17, 0.12]	-0.36	0.719	0.899	0.001	0.001

Table S9A. Summary of model results testing the interaction effect of RADS-2 total scores and socioeconomic disadvantage percentiles on fractional anisotropy (FA) for arcuate fasciculus (AF) and uncinate fasciculus (UF). In all linear models, age, sex, body mass index, psychiatric medication use, depression severity, Tanner stage, study group, race, scan time point, tract length, and motion during the scan, were included as covariates. All reported beta coefficients are standardized. CI=confidence interval; SE=standard error.

**Tract**	**Beta Coefficient**	**SE**	**95% CI**	***t*-value**	***p*-value**	**FDR-corrected *p*-value**	R^2	ΔR^2
Poverty Disadvantage Percentile								
L AF FA	0.13	0.07	[-0.01, 0.28]	1.82	0.070	0.140	0.208	0.021
L UF FA	0.06	0.07	[-0.09, 0.20]	0.76	0.448	0.448	0.200	0.004
R AF FA	0.06	0.09	[-0.12, 0.23]	0.63	0.527	0.527	0.098	0.003
R UF FA	-0.09	0.08	[-0.24, 0.06]	-1.16	0.249	0.498	0.152	0.008
Housing Burden Percentile								
L AF FA	0.06	0.08	[-0.10, 0.23]	0.77	0.442	0.442	0.160	0.004
L UF FA	0.09	0.08	[-0.07, 0.26]	1.10	0.272	0.442	0.162	0.008
R AF FA	0.07	0.09	[-0.11, 0.25]	0.78	0.437	0.437	0.090	0.004
R UF FA	-0.09	0.09	[-0.26, 0.08]	-1.06	0.292	0.437	0.114	0.007
Linguistic Isolation Percentile								
L AF FA	0.06	0.07	[-0.08, 0.20]	0.80	0.424	0.848	0.169	0.004
L UF FA	0.01	0.07	[-0.13, 0.16]	0.18	0.859	0.859	0.134	0.002
R AF FA	-0.05	0.08	[-0.21, 0.11]	-0.67	0.507	0.507	0.074	0.003
R UF FA	-0.07	0.08	[-0.22, 0.07]	-0.99	0.323	0.507	0.081	0.006

Table S9B. Summary of model results testing the interaction effect of RADS-2 total scores and socioeconomic disadvantage percentiles on fractional anisotropy (FA) for arcuate fasciculus (AF) and uncinate fasciculus (UF). All linear models are reported without covariates. All reported beta coefficients are standardized. CI=confidence interval; SE=standard error.

Tract	Beta Coefficient	SE	95% CI	*t*-value	*p*-value	FDR-corrected *p*-value	R^2	ΔR^2
Community Disadvantage Percentile								
L AF FA	0.15	0.07	[0.02, 0.29]	2.30	0.023*	0.046*	0.064	0.03
L UF FA	0.05	0.07	[-0.08, 0.19]	0.78	0.438	0.438	0.036	0.003
R AF FA	0.11	0.07	[-0.03, 0.25]	1.59	0.115	0.230	0.048	0.02
R UF FA	-0.03	0.07	[-0.17, 0.10]	-0.50	0.615	0.615	0.54	0.002
Education Percentile								
L AF FA	0.08	0.07	[-0.05, 0.22]	1.20	0.230	0.460	0.044	0.008
L UF FA	0.04	0.07	[-0.10, 0.18]	0.57	0.572	0.572	0.029	0.002
R AF FA	0.05	0.08	[-0.10, 0.20]	0.65	0.516	0.539	0.044	0.003
R UF FA	-0.04	0.07	[-0.18, 0.10]	-0.62	0.539	0.539	0.02	0.002
Unemployment Percentile								
L AF FA	0.18	0.07	[0.05, 0.32]	2.63	0.009**	0.018*	0.057	0.04
L UF FA	0.03	0.07	[-0.11, 0.17]	0.49	0.625	0.625	0.018	0.0013
R AF FA	0.09	0.07	[-0.05, 0.23]	1.22	0.225	0.450	0.024	0.009
R UF FA	-0.04	0.07	[-0.18, 0.10]	-0.59	0.556	0.556	0.02	0.002
Poverty Percentile								
L AF FA	0.14	0.07	[0.00, 0.27]	1.96	0.052	0.104	0.054	0.020
L UF FA	0.10	0.07	[-0.03, 0.24]	1.49	0.139	0.139	0.06	0.012
R AF FA	0.06	0.08	[-0.9, 0.22]	0.83	0.407	0.407	0.037	0.004
R UF FA	-0.08	0.07	[-0.22, 0.06]	-1.17	0.243	0.407	0.053	0.007
Housing Burden Percentile								
L AF FA	0.06	0.07	[-0.07, 0.20]	0.90	0.372	0.372	0.022	0.004
L UF FA	0.13	0.07	[-0.01, 0.27]	1.84	0.068	0.136	0.038	0.018

R AF FA	0.09	0.07	[-0.06, 0.23]	1.17	0.246	0.448	0.027	0.008
R UF FA	-0.05	0.07	[-0.19, 0.09]	-0.76	0.448	0.448	0.023	0.003
Linguistic Isolation Percentile								
L AF FA	0.004	0.07	[-0.13, 0.13]	-0.05	0.958	0.958	0.042	0.00002
L UF FA	0.02	0.07	[-0.11, 0.16]	0.33	0.739	0.958	0.013	0.0006
R AF FA	-0.05	0.07	[-0.19, 0.09]	-0.70	0.483	0.483	0.029	0.003
R UF FA	-0.11	0.07	[-0.24, 0.02]	-1.62	0.106	0.212	0.018	0.014

Table S9C. Summary of estimated linear regression of interaction effect of RADS-2 total scores and disadvantage percentiles on fractional anisotropy (FA) for arcuate fasciculus (AF) and uncinate fasciculus (UF) from CalEnviroScreen4.0 data. In all linear models, age, sex, body mass index, psychiatric medication use, depression severity, Tanner stage, study group, race, scan time point, tract length, and motion during the scan, were included as covariates. Socioeconomic disadvantage percentiles extracted from CalEnviroScreen 4.0. All reported beta coefficients are standardized. CI=confidence interval; SE=standard error.

**Tract**	**Beta Coefficient**	**SE**	**95% CI**	***t*-value**	***p*-value**	**FDR-corrected *p*-value**	R^2	ΔR^2
Community Disadvantage Percentile								
L AF FA	0.18	0.07	[0.04, 0.32]	2.57	0.011*	0.022*	0.205	0.041
L UF FA	0.02	0.07	[-0.12, 0.16]	0.29	0.770	0.770	0.169	0.00054
R AF FA	0.10	0.08	[-0.06, 0.26]	1.26	0.209	0.418	0.104	0.0116
R UF FA	-0.05	0.07	[-0.19, 0.10]	-0.64	0.524	0.524	0.135	0.0026
Education Percentile								
L AF FA	0.15	0.07	[0.02, 0.29]	2.19	0.03*	0.06	0.203	0.03
L UF FA	0.02	0.07	[-0.13, 0.16]	0.21	0.831	0.831	0.152	0.0003
R AF FA	0.02	0.08	[-0.14, 0.18]	0.22	0.825	0.825	0.105	0.0004
R UF FA	-0.07	0.07	[-0.22, 0.08]	-0.94	0.350	0.7	0.108	0.006

References

1. Ho, T. C., Sisk, L. M., Kulla, A., Teresi, G. I., Hansen, M. M., Wu, H., & Gotlib, I. H. (2021). Sex differences in myelin content of white matter tracts in adolescents with depression. Neuropsychopharmacology : official publication of the American College of Neuropsychopharmacology, 46(13), 2295–2303. https://doi.org/10.1038/s41386-021-01078-3

2. Ho, T. C., Kulla, A., Teresi, G. I., Sisk, L. M., Rosenberg-Hasson, Y., Maecker, H. T., & Gotlib, I. H. (2022). Inflammatory cytokines and callosal white matter microstructure in adolescents. Brain, behavior, and immunity, 100, 321–331. https://doi.org/10.1016/j.bbi.2021.12.003

3. Walker, J. C., Teresi, G. I., Weisenburger, R. L., Segarra, J. R., Ojha, A., Kulla, A., ... & Ho, T. C. (2020). Study protocol for teen inflammation glutamate emotion research (TIGER). Frontiers in Human Neuroscience, 14, 414.

4. Poznanski, E. O., & Mokros, H. B. (1996). Children's depression rating scale, revised (CDRS-R). Los Angeles: Western Psychological Services.

5. King, L. S., Humphreys, K. L., Camacho, M. C., & Gotlib, I. H. (2019). A person-centered approach to the assessment of early life stress: Associations with the volume of stress-sensitive brain regions in early adolescence. Development and psychopathology, 31(2), 643–655. https://doi.org/10.1017/S0954579418000184

6. Chahal, R., Miller, J. G., Yuan, J. P., Buthmann, J. L., & Gotlib, I. H. (2022). An exploration of dimensions of early adversity and the development of functional brain network connectivity during adolescence: Implications for trajectories of internalizing symptoms. Development and psychopathology, 34(2), 557–571. https://doi.org/10.1017/S0954579421001814

7. Yeatman, J. D., Dougherty, R. F., Myall, N. J., Wandell, B. A., & Feldman, H. M. (2012). Tract profiles of white matter properties: automating fiber-tract quantification. PloS one, 7(11).

8. Ho, T. C., King, L. S., Leong, J. K., Colich, N. L., Humphreys, K. L., Ordaz, S. J., & Gotlib, I. H. (2017). Effects of sensitivity to life stress on uncinate fasciculus segments in early adolescence. Social Cognitive and Affective Neuroscience, 12(9), 1460-1469. doi:10.1093/scan/nsx065

9. Ho, T. C., Teresi, G. I., Segarra, J. R., Ojha, A., Walker, J. C., Gu, M., Spielman, D. M., Sacchet, M. D., Jiang, F., Rosenberg-Hasson, Y., Maecker, H., & Gotlib, I. H. (2021). Higher Levels of Pro-inflammatory Cytokines Are Associated With Higher Levels of Glutamate in the Anterior Cingulate Cortex in Depressed Adolescents. Frontiers in psychiatry, 12, 642976. https://doi.org/10.3389/fpsyt.2021.642976

10. Wakana, S., Caprihan, A., Panzenboeck, M. M., Fallon, J. H., Perry, M., Gollub, R. L., ... & Blitz, A. (2007). Reproducibility of quantitative tractography methods applied to cerebral white matter. Neuroimage, 36(3), 630-644.

11. Mori, S., Kaufmann, W. E., Davatzikos, C., Stieltjes, B., Amodei, L., Fredericksen, K., ... & Moser, H. W. (2002). Imaging cortical association tracts in the human brain using diffusion-tensor-based axonal tracking. Magnetic Resonance in Medicine: An Official Journal of the International Society for Magnetic Resonance in Medicine, 47(2), 215-223.

12. Kircanski, K., Sisk, L. M., Ho, T. C., Humphreys, K. L., King, L. S., Colich, N. L., ... & Gotlib, I. H. (2019). Early life stress, cortisol, frontolimbic connectivity, and depressive symptoms during puberty. Development and psychopathology, 31(3), 1011-1022.
